# A systematic review of ethnobotanical study in Indonesia: diversity and cultural patterns of medicinal plant use

**DOI:** 10.1186/s13002-026-00879-4

**Published:** 2026-03-16

**Authors:** Raden M. Febriyanti, Zelika M. Ramadhania, Ami Tjitraresmi, Raden B. Indradi, Intan T. Maisyarah, Patrick M. Maundu, Muhaimin Muhaimin, Wawan Sujarwo

**Affiliations:** 1 Department of Biological Pharmacy, Faculty of Pharmacy, Universitas Padjadjaran, Bandung, Indonesia; 2 Herbal Study Center, Universitas Padjadjaran, Bandung, Indonesia; 3 Kenya Resource Centre for Indigenous Knowledge (KENRIK), Kenya National Museum, Nairobi, Kenya; 4 Research Center for Ecology, National Research and Innovation Agency, Bogor, Indonesia

**Keywords:** Indonesia, Ehnobotany, Medicinal plants, Traditional medicine, Informant consensus factor

## Abstract

**Background:**

Indonesia holds immense biocultural diversity, yet ethnobotanical data remain fragmented. This systematic review synthesizes medicinal plant use across the archipelago to identify cross-cultural patterns, define a core ethnomedicinal flora, and critically appraise methodological rigor.

**Methods:**

In adherence to PRISMA 2020 guidelines, primary field studies were retrieved from PubMed, Scopus, and GARUDA. Scientific names were harmonized using *Plants of the World Online* (POWO), and therapeutic indications were mapped to ICPC-2 disease categories. Methodological quality was assessed using a modified JBI checklist.

**Results:**

A total of 91 studies were analyzed, yielding 3,202 use-reports for 791 medicinal plant species. Documentation was found to be geographically skewed toward Sumatra and Java, leaving Eastern Indonesia significantly underrepresented. A shared medicinal flora of 239 species was identified across multiple ethnic groups, dominated by versatile plants such as *Zingiber officinale* and *Orthosiphon aristatus*. High consensus was observed for acute conditions (e.g., *Psidium guajava* for diarrhoea), whereas chronic diseases like hypertension demonstrated diverse plant utilization. Quality assessment revealed that 89% of the included studies lacked explicit ethics reporting, and 32% relied on secondary identification without voucher specimens.

**Conclusions:**

A culturally salient core of medicinal plants was identified alongside unique regional adaptations. However, the existing literature is compromised by weak taxonomic validation and non-standardized reporting. Future research priorities must include the documentation of neglected regions and the enforcement of rigorous, voucher-based methodologies.

**Supplementary Information:**

The online version contains supplementary material available at 10.1186/s13002-026-00879-4.

## Introduction

Ethnobiology and ethnobotany are increasingly acknowledged as vital disciplines for conserving traditional knowledge systems, which face imminent erosion from global environmental change, urbanization, and language shift [1, 2]. Beyond their archival value, these disciplines provide the empirical basis for understanding how communities adapt health practices to local environments, preserving a biocultural heritage that might otherwise be eroded by modernization [3, 4]. Simultaneously, ethnobotanical knowledge has long underpinned drug discovery and phytomedicine development. Historical analyses consistently demonstrate that a substantial proportion of approved drugs originate from natural products initially identified through traditional medical systems [5–7]. By leveraging ethnobotanical data, ethnopharmacology prioritizes species for mechanistic investigation, thereby narrowing the search space and improving the efficiency of bioprospecting [8, 9]. Ethnobotanical research is also central to conservation planning because it identifies which species and habitats are most salient to local health systems and livelihoods [10, 11].

Indonesia, as a megadiverse country, offers a unique landscape for examining these biocultural dynamics. The archipelago harbours exceptionally high plant diversity, home to an estimated 30,000 to 40,000 species of flowering plants [12]. This biological richness coincides with profound ethnographic diversity. The nation comprises approximately 713 ethnic groups and 360–719 language groups across the archipelago, with Bahasa Indonesia serving as the official unifying language [13, 14]. This intersection of nature and culture has fostered a vibrant traditional medicinal heritage that spans preventive, curative, and ritual domains. Case studies ranging from the Batak Karo in North Sumatra to Balinese *usada* traditions demonstrate that plant-based therapies remain deeply embedded in everyday health-seeking behavior [15–19]. Yet, this biodiversity faces significant pressures. Quantitative gap analyses indicate that many priority medicinal species are located outside protected-area networks and are threatened by land-use conversion and climate change [12, 20]. Consequently, strategic conservation depends not only on species distribution models but also on a systematic synthesizes of how different communities value and manage these taxa [8, 12].

Despite the abundance of local ethnobotanical surveys such as those among the Tengger, To Manui, and Dayak Desa communities, the national data landscape remains fragmented [18, 19, 21]. While these studies have successfully catalogued local plant indications and often employ indices like Use Value (UV) or Relative Frequency of Citation (RFC) [17, 22] they vary widely in sampling strategies, taxonomic verification, and disease classification [11, 23]. Existing reviews tend to be either thematic (e.g., focusing solely on antidiabetic plants) or geographically restricted, failing to provide a harmonized, cross-ethnic analysis of the Indonesian pharmacopoeia [21, 22, 24, 25]. Furthermore, international methodological critiques highlight that many such studies remain descriptive and inconsistently report vouchers, ethics, and statistical indices underscoring the need for more rigorous, theory-informed synthesis [26–28].

In this systematic review, primary ethnobotanical surveys among Indonesian ethnic groups were synthesized to bridge existing data gaps. Plant names were reconciled to accepted species name and reported ailments were mapped to standard ICPC-2 disease categories. Additionally, the cross-cultural prominence of medicinal plants was quantified across diverse ethnic groups and regions. Species and locations requiring prioritized biocultural conservation and pharmacological investigation were identified, alongside a systematic evaluation of deficiencies in voucher specimens, ethical consent reporting, and methodological rigor.

## Methods

### Search strategy and data sources

This systematic review was conducted in accordance with the Preferred Reporting Items for Systematic Reviews and Meta-Analyses (PRISMA 2020) statement, following a protocol registered in PROSPERO (CRD420251136256; available at: https://www.crd.york.ac.uk/PROSPERO/view/CRD420251136256). To ensure a comprehensive synthesis of Indonesian ethnobotany, a systematic search was undertaken in three major electronic databases: PubMed, Scopus, and the Garba Rujukan Digital Indonesia (GARUDA) portal. The GARUDA portal was included to capture regionally published studies and grey literature that are frequently underrepresented in global indexing services. In addition, reference lists of included articles were manually screened to identify further eligible sources not retrieved through the electronic search. The search strategy was constructed using a combination of controlled vocabulary (MeSH terms) and free-text keywords structured around three core concepts: (1) discipline (e.g., “ethnobotany,” “ethnomedicine,” “ethnopharmacology,” “traditional medicine”); (2) subject (e.g., “medicinal plants,” “herbal medicine,” “indigenous knowledge”); and (3) geography (e.g., “Indonesia,” including island regions such as Java, Sumatra, Kalimantan, Sulawesi, and Papua). To minimize language bias and maximize retrieval of local literature, all terms were applied in both English and Bahasa Indonesia (e.g., “etnobotani,” “obat tradisional,” “tanaman obat”).

### Inclusion and exclusion criteria

Eligibility criteria were defined in the PROSPERO protocol and were applied during the screening process. Included studies were primary field-based ethnobotanical or ethnomedicinal surveys conducted in Indonesia involving human informants (community members, traditional healers, birth attendants, elders, caregivers) in community or primary-care settings, regardless of age or sex. Eligible designs comprised cross-sectional ethnobotanical surveys and qualitative or mixed-methods studies that used interviews, free-listing, focus groups, participant observation, participatory rural appraisal, or similar methods to document health-related medicinal use of plants. Studies based solely on literature compilations, herbarium labels, clinical records, or laboratory experiments without primary community data were excluded, as were purely phytochemical or pharmacological studies lacking explicit ethnobotanical context. To be included, studies were required to report medicinal uses of plants with therapeutic intent and to provide at least genus-level identification, preferably to species, supported by a verifiable method such as voucher deposition, expert taxonomic determination, or use of standard floras and plant databases. Studies were required to provide extractable medicinal-use data, including reported ailments or therapeutic claims that could be mapped to standard health categories. Peer-reviewed articles and theses with full primary methods were eligible, whereas reviews, editorials, conference abstracts, and duplicate reports were excluded, with the most complete version retained in cases of overlapping datasets.

### Study selection and quality assessment

All retrieved records were imported into reference management software, and duplicates were removed. The screening process was conducted in two stages using Rayyan (rayyan.ai). First, titles and abstracts were screened independently by two reviewers (RMF and RBI) against the eligibility criteria. Second, potentially relevant full-text articles were retrieved and assessed for final inclusion. Disagreements were resolved through consensus or consultation with a third reviewer (ITM).

**Fig. 1:**
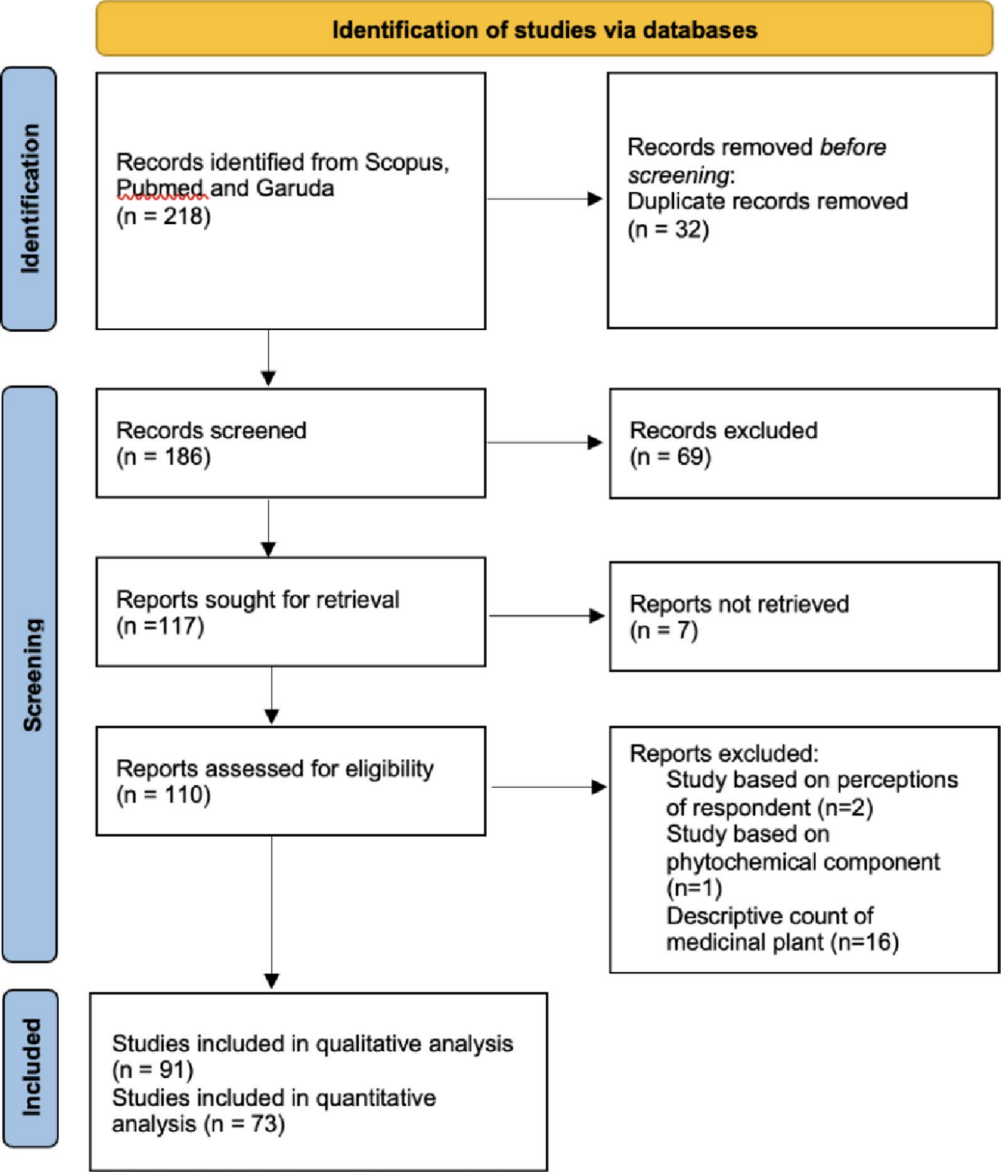
PRISMA flow diagram

The methodological quality of the included studies was appraised using the JBI Critical Appraisal Checklist for Analytical Cross-Sectional Studies [2], adapted to incorporate ethnobotany-specific indicators. These indicators included the definition of use-reports, clarity of taxonomic verification, reporting of voucher specimens, and the completeness of ailment reporting. Quality assessment scores did not constitute an exclusionary threshold; rather, this appraisal was employed to contextualize the findings and evaluate the methodological rigor of the dataset. The systematic search and selection process is detailed in the PRISMA flow diagram (Fig. 1). Data extraction was performed by one reviewer using a standardized form and cross-checked by a second reviewer. Extracted variables included bibliographic details, geographic context (province, island region), cultural context (ethnic group), and methodological characteristics (sampling design, informant demographics).

For each recorded plant, we extracted the reported scientific name, local name, part used, mode of preparation, and route of administration. A critical step in this review was taxonomic harmonization. All reported scientific names were cross-referenced against *Plants of the World Online* (POWO) to validate taxonomy, correct synonyms to accepted names, and ensure standardized reporting. Reported ailments were mapped to standardized health categories using the International Classification of Primary Care, second edition (ICPC-2), to facilitate cross-study comparison.

### Data analysis

Given the methodological variations among the included studies, a narrative synthesis was conducted, complemented by descriptive statistics. Cultural importance and consensus were quantified by calculating Use Reports (UR). Following the approach of Meniza et al. (2024), each study was treated as a single ‘informant’ unit to enable data harmonization. Cross-ethnic prominence was assessed by determining the number of ethnic groups and regions citing each entry, through which widely used core species were identified [29].

## Results

### Overview and quality appraisal of the included studies

**Fig. 2:**
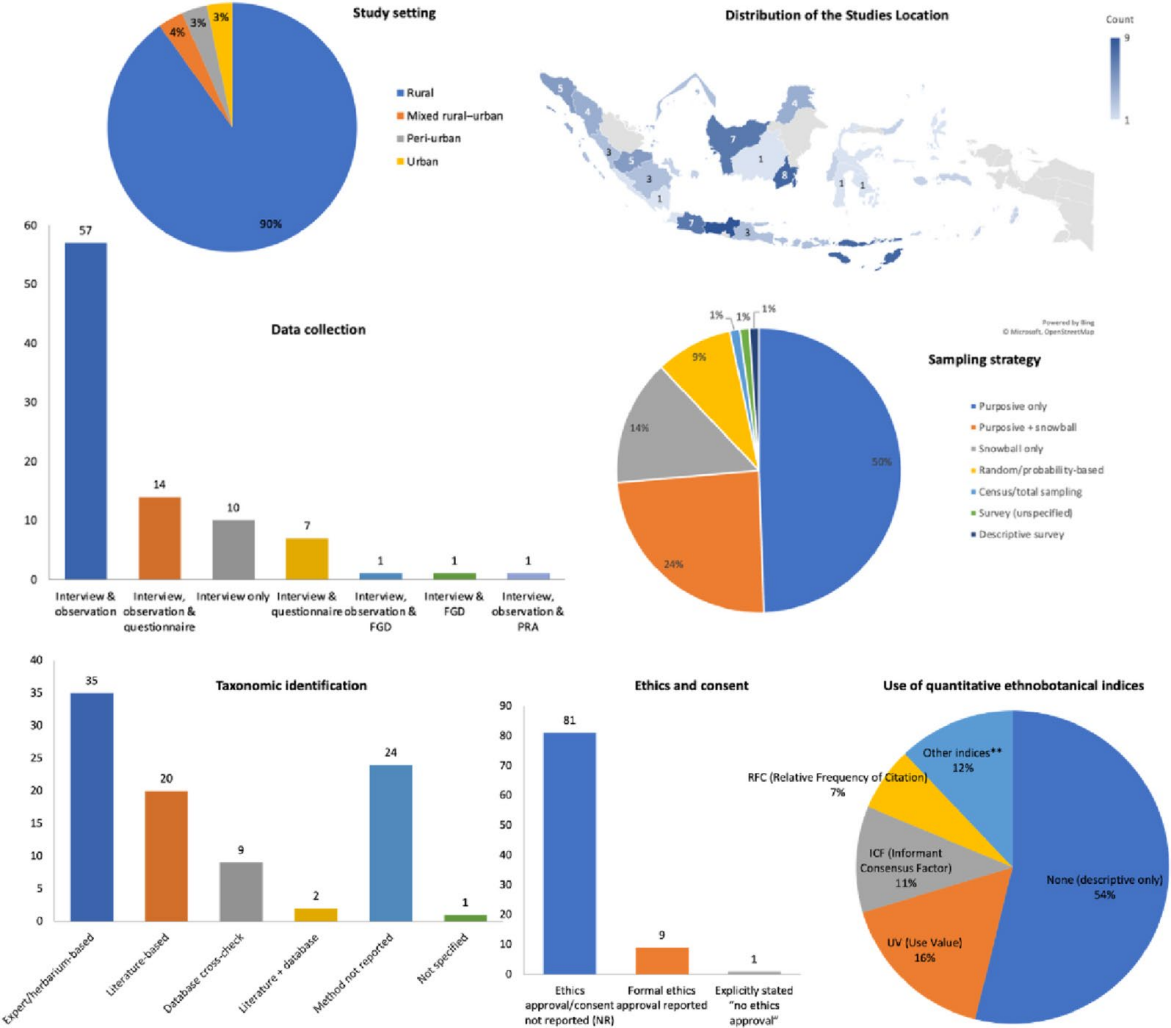
Descriptive analysis of the included studies

A total of 91 primary ethnobotanical field studies were identified for synthesis and their descriptive characteristics are provided in Supplementary File 1, Table S1. Summary of Included Studies and Table S2. Characteristics of Included Studies [16, 18, 21, 30–116]. Descriptive analysis across the 91 included studies (Fig. 2) indicates a strong geographic concentration in Sumatra (28.6%) and most studies were conducted in rural settings (90.1%). Methodologically, sampling was dominated by non-probability approaches, particularly purposive sampling alone (49.5%) while data acquisition primarily relied on interviews coupled with observation (62.6%). Taxonomic verification most often used expert/herbarium identification (38.5%). Reporting of research governance was limited with ethics approval or consent was not reported in 89.0% of studies. Quantitative ethnobotanical inference was also frequently absent, with over half reporting no indices (53.8%).

**Fig. 3:**
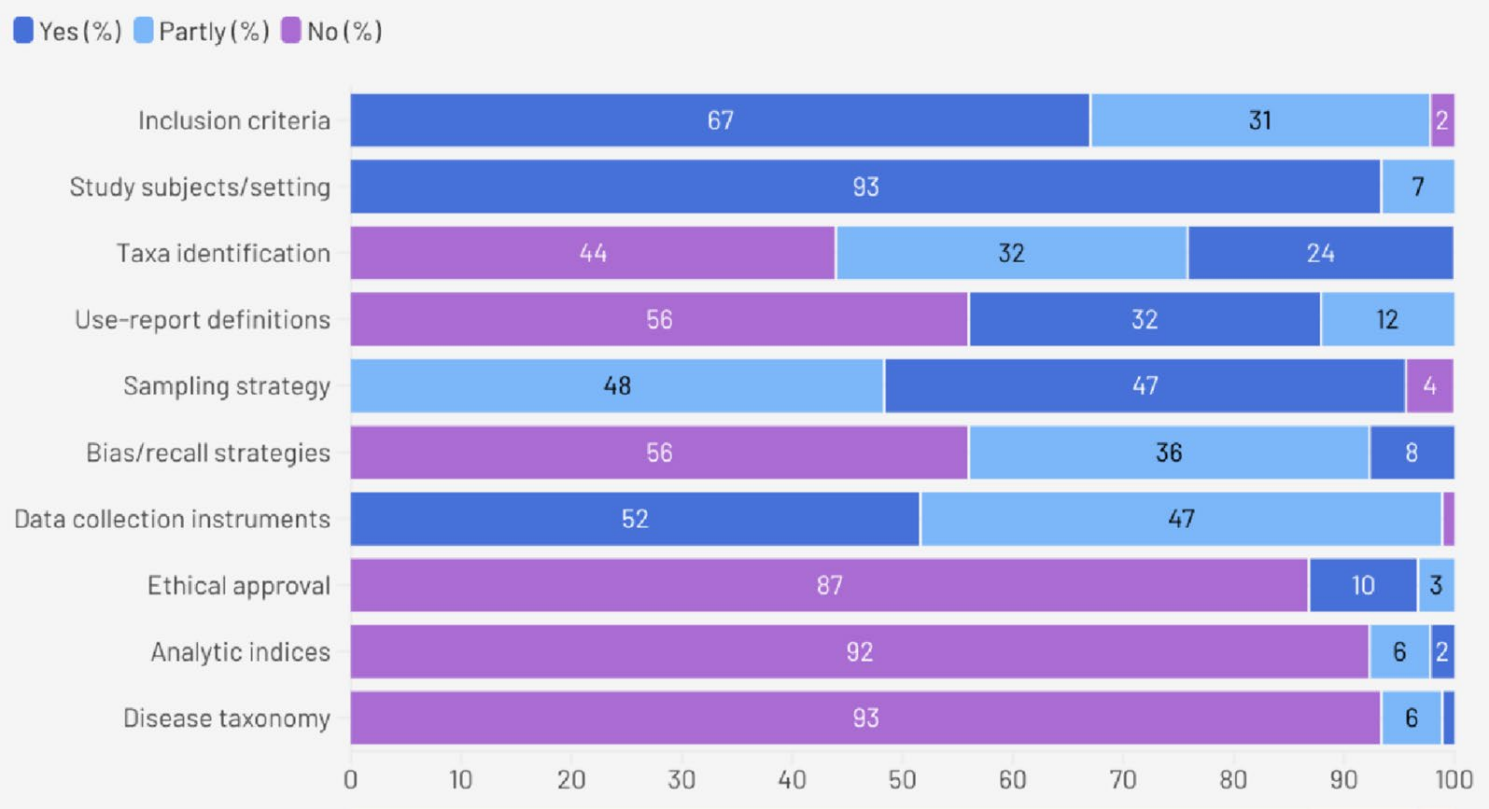
Quality appraisal of the 91 included ethnobotanical studies

Furthermore, the methodological quality of the 91 included studies was evaluated using a ten-item checklist, with a maximum possible score of 10. The overall quality of the dataset was moderate with scores ranged from a minimum of 1.5 to a maximum of 7.5 (Supplementary File S2 - Quality Appraisal of the Included Studies). The distribution of total scores is depicted in Fig. 3.

Notably, 93.4% of studies provided detailed descriptions of study subjects and settings and 67.0% clearly defined inclusion criteria for participants. However, significant gaps were observed in ethical reporting and standardization. Only 9.9% of studies explicitly reported obtaining ethical approval or informed consent, with the vast majority (86.8%) failing to mention these protocols. Similarly, standardization of medical terminology was largely absent. 93.4% of studies failed to map ailments to a standard disease taxonomy such as the ICD or ICPC. Furthermore, quantitative rigor was often lacking, as 92.3% of studies did not present clear analytic methods for indices such as Informant Consensus Factor (ICF) or Use Value (UV). While 51.6% of studies adequately described their data collection instruments, strategies to address bias and recall such as triangulation or prompts were absent in 56.0% of the literature.

### Geographical distribution and cross ethnicity prominence

**Fig. 4:**
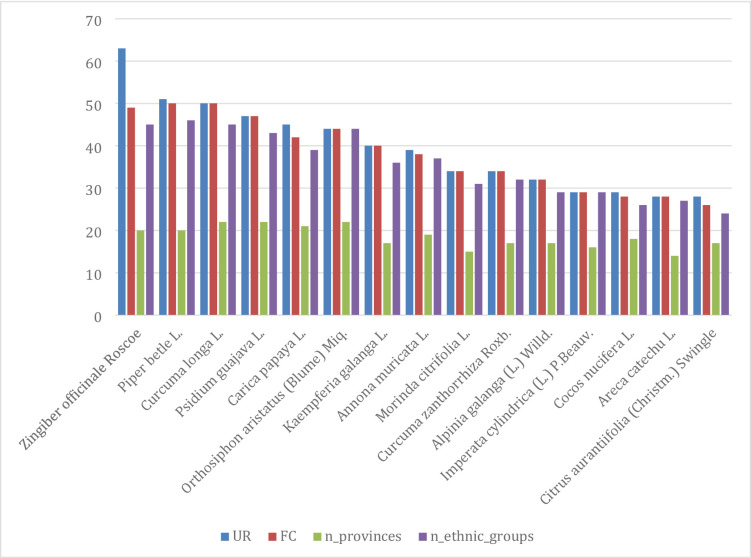
Use Reports of 15 most reported medicinal plants across province and ethnicity

Use-reports of medicinal plants were documented from 73 ethnobotanical studies, yielding 3,202 individual use-reports for 791 plant species across 24 Indonesian provinces (Supplementary File S3 – Use Reports of Medicinal Plants). Analysis of cross-cultural distribution revealed that 239 species were recorded across multiple provinces and ethnic groups, whereas the majority of the remaining species were locally restricted. The geographical prominence of the 15 most frequently cited species is visualized in Fig. 4, which highlights the widespread utilization of core medicinal plants such as *Zingiber officinale* and *Piper betle* across diverse provincial landscapes.

**Fig. 5:**
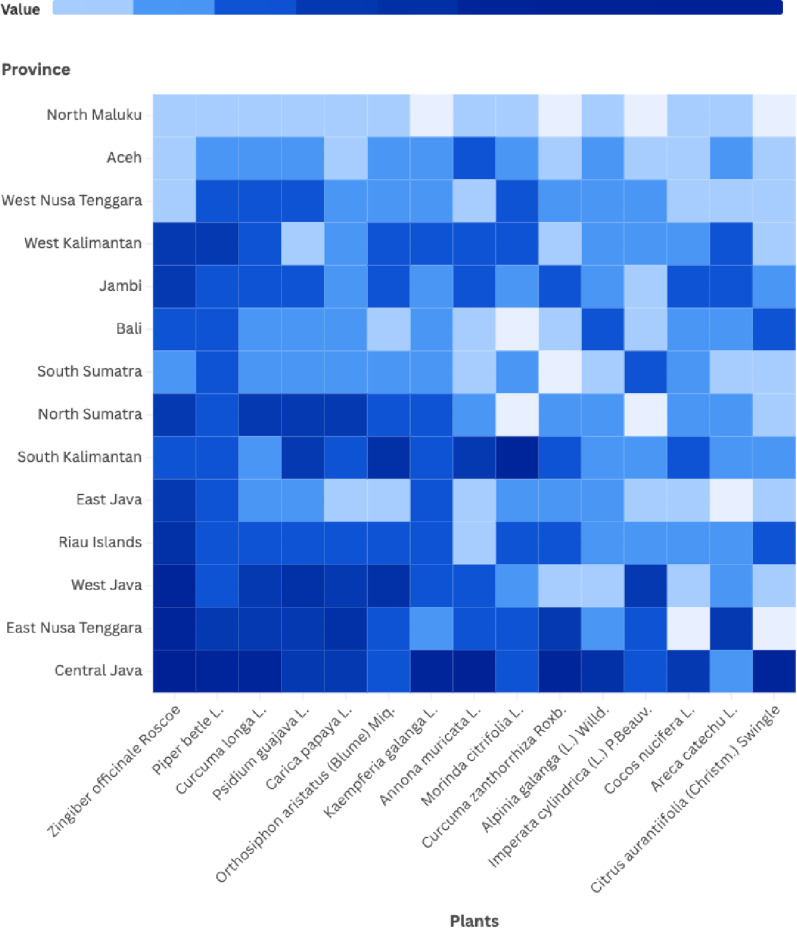
The heatmap of use-reports for 15 most reported species

The heatmap of use-reports for these 15 most reported species across provinces further underscores both shared and regionally nuanced patterns. Central Java, West Java, North Sumatra, West and South Kalimantan, East and West Nusa Tenggara, Bali and the Riau Islands exhibit consistently high intensities for many of these species, reflecting dense and diversified medicinal use (Fig. 5).

### Identification of plants with high versatility across various ailments

**Fig. 6:**
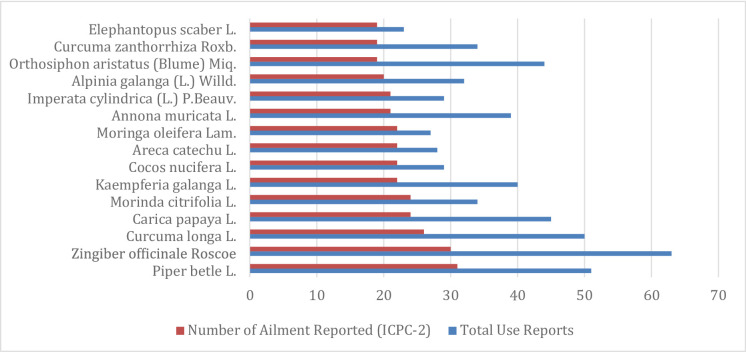
Most versatile medicinal plant species across ICPC-2 ailment categories

This study identified 3,202 use-reports of 791 medicinal plant species across 163 ICPC-2 ailment categories (Supplementary File S4 - Use Reports of Medicinal Plants Across ICPC-2 Mapped Ailments). While most species were cited for a single ailment category, a subset demonstrated significant versatility. Figure 6 illustrates the relationship between citation frequency and therapeutic range. *Piper betle* L. exhibited the broadest utility, treating over 30 distinct ICPC-2 categories followed by *Zingiber officinale* Roscoe, *Curcuma longa* L., and *Carica papaya* L.

Table 1 details the 15 most frequently reported ailment mapped to ICPC-2 codes. Fever, uncomplicated hypertension, type 2 diabetes, cough, and diarrhoea were the most common complaints. For metabolic and cardiovascular disease, while the conditions are frequently reported, plant use was diffuse with no single species dominating. For instance, in hypertension, *Apium graveolen*s L. contributed only 6% of reports, and for type 2 diabetes, *Muntingia calabura* L. accounted for approximately 4.5%. Conversely, specific conditions demonstrated clear species dominance. *Orthosiphon aristatus* (Blume) Miq. accounted for 24% of reports for kidney stone, confirming its status as a primary diuretic. *Psidium guajava* L. was the principal remedy for diarrhoea, while *Carica papaya* L. led for malaria. *Syzygium polyanthum* (Wight) Walp. and *Annona muricata* L. were frequently cited for lipid disorders (and malignancy. Notably, *Muntingia calabura* L. and *Zingiber officinale* Roscoe recurred across multiple categories, mirroring their high rankings in Fig. 6.

**Table 1:** Fifteen most frequently reported ICPC-2 ailments and their most frequently cited medicinal plant species

No.	Ailments (ICPC-2 mapped)	Total UR	Top 5 plant species (number of UR within that ailment)
1	Fever	267	*Kalanchoe pinnata* (Lam.) Pers. (9 UR); *Hibiscus rosa-sinensis* L. (6 UR); *Andrographis paniculata* (Burm.f.) Wall. ex Nees (6 UR); *Ceiba pentandra* (L.) Gaertn. (6 UR); *Jatropha curcas* L. (6 UR)
2	Hypertension, uncomplicated	158	*Apium graveolens* L. (9 UR); *Persea americana* Mill. (7 UR); *Syzygium polyanthum* (Wight) Walp. (5 UR); *Averrhoa bilimbi* L. (5 UR); *Physalis angulata* L. (5 UR)
3	Type 2 diabetes (non-insulin-dependent)	155	*Muntingia calabura* L. (7 UR); *Orthosiphon aristatus* (Blume) Miq. (5 UR); *Physalis angulata* L. (5 UR); *Persea americana* Mill. (4 UR); *Carica papaya* L. (3 UR)
4	Cough	145	*Foeniculum vulgare* Mill. (6 UR); *Zingiber officinale* Roscoe (6 UR); *Muntingia calabura* L. (5 UR); *Allium cepa* L. (4 UR); *Curcuma longa* L. (4 UR)
5	Laceration or skin cut	120	*Stachytarpheta jamaicensis* (L.) Vahl (5 UR); *Muntingia calabura* L. (4 UR); *Kalanchoe pinnata* (Lam.) Pers. (3 UR); *Chromolaena odorata* (L.) R.M. King & H. Rob. (3 UR); *Psidium guajava* L. (3 UR)
6	Diarrhoea	120	*Psidium guajava* L. (10 UR); *Languas galanga* (L.) Stuntz (3 UR); *Morinda citrifolia* L. (3 UR); *Orthosiphon aristatus* (Blume) Miq. (3 UR); *Citrus aurantiifolia* (Christm.) Swingle (3 UR)
7	General symptom (non-specific pain)	95	*Orthosiphon aristatus* (Blume) Miq. (7 UR); *Citrus aurantiifolia* (Christm.) Swingle (6 UR); *Annona muricata* L. (6 UR); *Persea americana* Mill. (5 UR); *Muntingia calabura* L. (5 UR)
8	Abdominal pain/cramps, general	93	*Muntingia calabura* L. (3 UR); *Psidium guajava* L. (3 UR); *Annona muricata* L. (3 UR); *Languas galanga* (L.) Stuntz (3 UR); *Ageratum conyzoides* L. (2 UR)
9	Stomach function disorder	73	*Foeniculum vulgare* Mill. (5 UR); *Muntingia calabura* L. (4 UR); *Curcuma longa* L. (3 UR); *Annona muricata* L. (3 UR); *Zingiber officinale* Roscoe (3 UR)
10	Joint symptoms	68	*Muntingia calabura* L. (3 UR); *Curcuma longa* L. (3 UR); *Moringa oleifera* Lam. (3 UR); *Annona muricata* L. (2 UR); *Morinda citrifolia* L. (2 UR)
11	Malaria	67	*Carica papaya* L. (5 UR); *Physalis angulata* L. (4 UR); *Persea americana* Mill. (3 UR); *Artemisia annua* L. (3 UR); *Annona muricata* L. (3 UR)
12	Teeth or gum complaint	60	*Muntingia calabura* L. (3 UR); *Annona muricata* L. (3 UR); *Foeniculum vulgare* Mill. (2 UR); *Syzygium aromaticum* (L.) Merr. & L.M. Perry (2 UR); *Zingiber officinale* Roscoe (2 UR)
13	Malignancy	57	*Annona muricata* L. (4 UR); *Zingiber officinale* Roscoe (3 UR); *Curcuma longa* L. (3 UR); *Muntingia calabura* L. (3 UR); *Morinda citrifolia* L. (2 UR)
14	Kidney stone	55	*Orthosiphon aristatus* (Blume) Miq. (13 UR); *Strobilanthes crispa* (L.) Blume (6 UR); *Phyllanthus urinaria* L. (4 UR); *Zingiber officinale* Roscoe (2 UR); *Imperata cylindrica* (L.) P. Beauv. (2 UR)
15	Lipid disorder	48	*Syzygium polyanthum* (Wight) Walp. (4 UR); *Morinda citrifolia* L. (4 UR); *Annona muricata* L. (3 UR); *Moringa oleifera* Lam. (3 UR); *Alpinia galanga* (L.) Willd. (2 UR)

**Fig. 7:**
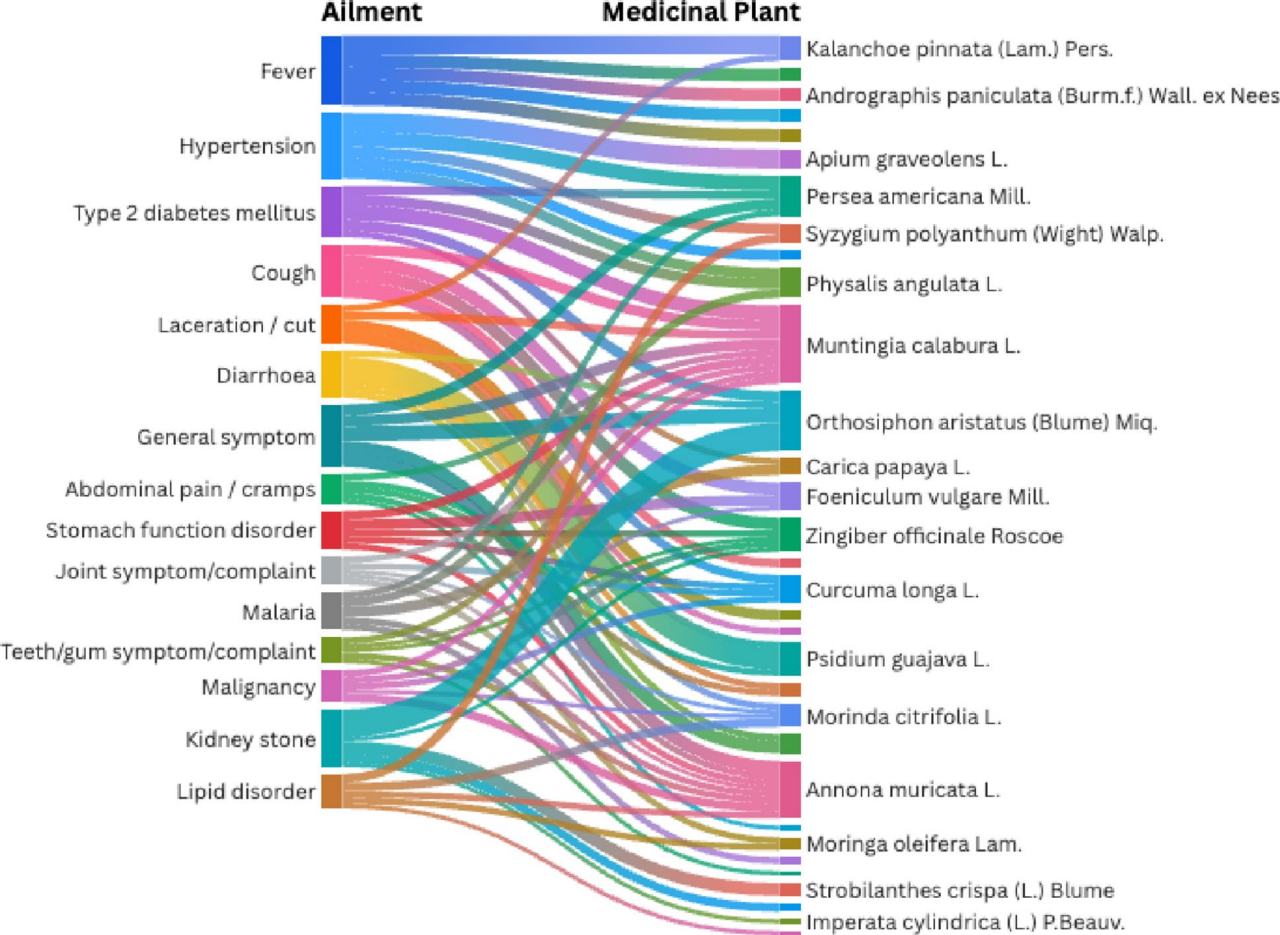
Sankey diagram linking major ICPC-2 ailment categories to the 18 most frequently cited medicinal plant species

The Sankey diagram (Fig. 7) visualizes the complex network connecting reported ailment categories to the 18 most frequently cited medicinal plant species. The width of each stream is proportional to the volume of use-reports, with thicker flows indicating higher citation frequency. Overall, the diagram reveals a highly interconnected medicinal system, characterized by a pattern where ailments are managed through diverse species and individual plants are employed for multiple therapeutic indications.

Distinct patterns of versatility are evident. *Muntingia calabura* L., *Orthosiphon aristatus* (Blume) Miq., and *Annona muricata* L. emerge as polyvalent core remedies, exhibiting prominent flows linking them to fever, hypertension, type 2 diabetes mellitus, general symptoms, abdominal pain, kidney stones, and lipid disorders. Consequently, these species function as versatile multi-purpose agents within the local medicinal plant, particularly for systemic and chronic conditions. A second cluster, comprising *Zingiber officinale* Roscoe, *Curcuma longa* L., *Foeniculum vulgare* Mill., *Carica papaya* L., and *Psidium guajava* L., demonstrates strong linkages to gastrointestinal and respiratory complaints, specifically abdominal pain, stomach function disorders, and diarrhoea, underscoring their primary utility as digestive remedies.

## Discussion

### Ethnobotanical studies in Indonesia

Our quantitative synthesis confirms that Indonesian ethnobotanical knowledge has been documented in a markedly uneven way across the archipelago with Sumatra (28.6%), Kalimantan (24.2%), and Java (20.9%) accounting for the majority of publications. Study in Mindanao, Philippines likewise found 530 medicinal species but highlighted that ethnobotanical surveys were geographically concentrated and called explicitly for targeted work in under-sampled provinces [29]. Studies from the western Himalaya in India similarly focus on specific protected areas and tribal groups, including communities in the Nanda Devi Biosphere Reserve and in Kangra and Chamba districts, while other communities and landscapes remain poorly represented in the published record [117, 118]. Bibliometric and field studies on Iranian medicinal plants show that certain provinces and cultural groups have attracted more research attention than others, and that modernization and shifting lifestyles are contributing to the erosion of traditional knowledge where it is not actively recorded [119–121].

Furthermore, ethnobotanical research in Indonesia is firmly grounded in anthropological and ecological description. Comparable patterns are visible across Asia and Africa. Ethnobotanical surveys from North-eastern Algeria, Sidi Kacem in Morocco, the Seshachalam Biosphere Reserve in India, Acipayam in Turkey, and malaria-focused work in Kwara State, Nigeria all provide detailed descriptions of geography, vegetation, ethnic composition and respondent characteristics, often combined with careful documentation of local preparation methods and emic disease categories [122–125]. At the same time, many of these international studies share the same limitations that we observed in Indonesia regarding methodological rigor and reproducibility. Purposive sampling was the most prevalent approach (49.5%), often explicitly targeting cultural specialists such as village healers (dukun), ritual experts (balian), or homegarden stewards [47, 52]. This reliance on purposive sampling reflects the stratified nature of ethnomedical knowledge transmission in Indonesia, where expertise is often lineage-based, gendered, or role-specific. For instance, studies in Aceh and Sumatra frequently targeted midwives and elder women [42, 70, 81], while research among minority groups like the Bunaq and Dayak focused on shamans [16, 51, 77]. Consequently, sample sizes in these specialist-focused surveys were often small (≤ 10 informants) [47, 52, 77]. Similarly, national ethnobotanical research record that much of the literature remains largely descriptive, with limited use of probabilistic sampling, standardized diagnostic categories or advanced quantitative analysis [126, 127]. Deliberative methodologies such as Focus Group Discussions (FGD) or Participatory Rural Appraisal (PRA) were rare, indicating that Indonesian ethnobotany remains largely extractive rather than participatory [37, 95].

Our appraisal also reveals substantial weaknesses in taxonomic validation and use of ethnobotanical indices within Indonesian ethnobotanical research. Several studies relied on literature-based identification, citing regional floras such as Flora of Java or pharmacognostic texts like Atlas Tumbuhan Obat [39, 58], cross-referencing global databases (e.g., POWO, GBIF) or using image-based tools like PlantNet [34, 75]. This level of uncertainty directly undermines the reproducibility of pharmacological and phytochemical research, where precise species identity is essential for toxicity assessment, dose standardisation, and conservation planning [128, 129]. Ethnobotanical studies from Morocco likewise stress voucher-based identification yet note that many surveys in still depend heavily on vernacular names and do not consistently report their taxonomic procedures, creating ambiguity when different local names refer to the same species or, conversely, when the same name is used for multiple species [130]. The implications of weak taxonomic foundations extend well beyond field surveys. Reviews of African medicinal plants emphasise that misidentification or taxonomic ambiguity at the ethnobotanical stage can propagate errors into phytochemical databases thereby reducing the reliability of candidate selection and wasting scarce research resources [131].

The situation is similar for quantitative ethnobotanical indices. The global literature shows that rich qualitative descriptions of ailments and plant uses are rarely matched by systematic mapping to international disease taxonomies or by fully transparent quantitative analysis [132–135]. Even when indices such as Use Value or Informant Consensus Factor are calculated, they are not always accompanied by clear definitions, denominators or measures of uncertainty, which constrains comparability across sites [125, 136].

### Geographical distribution and cross ethnicity prominence

Analysis of cross-cultural distribution revealed that 239 species were recorded across multiple provinces and ethnic groups highlights the widespread utilization of medicinal plants such as *Zingiber officinale* and *Piper betle* across diverse provincial landscapes. In contrast to these widely shared medicinal plants, a distinct subset of species was found to be tightly linked to specific regions and ethnic groups. These highly localized species exemplify region- and ethnicity-specific ethnomedicinal practices, which may reflect unique therapeutic strategies and biocultural adaptations. For example, among the Dayak Meratus in South Kalimantan, *Tristaniopsis* sp. was reported for mouth/tongue/lip disease using water from the stem [74] and separately for hematuria using the bark [77]. In the same ethnoecological context, roots of *Artocarpus odoratissimus* was used for abdominal pain or cramps [77] and its leaves for uncomplicated hypertension [95]. In Java, fruit of *Medinilla speciosa* was recorded among Javanese communities in Central Java for female infertility [41] and in East Java for lactation-related complications [137], highlighting culturally embedded postpartum and reproductive health practices. Likewise, within Balinese communities in Bali, several species illustrated region-specific use. For example, leaves of *Casuarina junghuhniana* was used for psychological disorders [47] and its stem sap for diabetes mellitus [89]; *Curcuma purpurascens* rhizome was used for liver disease [47] and for asthma [89]; and stem and leaves of *Cryptocarya massoy* was reported for fracture-related conditions [47] as well as coordination disorders [89]. Collectively, these examples underscore that, alongside a widely shared medicinal plants use, Indonesia’s ethnopharmacological landscape contains culturally patterned plant–indication–part-used associations that likely arise from the interaction of local ecology, knowledge transmission, and culturally specific health priorities.

### Plants with high versatility across various ailments

our analysis finds that *Zingiber officinale*,* Curcuma longa*,* Carica papaya*,* Morinda citrifolia*,* Moringa oleifera* and *Orthosiphon aristatus* emerge as highly versatile taxa that are repeatedly cited in studies from different islands and used to treat a wide spectrum of complaints, including metabolic, digestive, infectious and general symptoms. Their wide spatial and therapeutic spread suggests that communities perceive these species as reliable, multi-purpose remedies, making them strong candidates for pharmacological prioritisation, safety assessment and nutraceutical development. This pattern of a shared, high-consensus medicinal plants align closely with ethnobotanical observations from other parts of Asia and Africa. In South Africa, an appraisal of antidiabetic plants in KwaZulu-Natal similarly highlights a subset of culturally important species including *Moringa oleifera* and other widely known species that dominate local treatment and are now being investigated phytochemically to bridge indigenous knowledge with modern healthcare [138].

Our analysis in most frequently reported ailments mapped to ICPC-2 categories reveals nuanced patterns in medicinal plant use, distinguishing between conditions with clear species dominance and those with heterogeneous treatment options. For gastrointestinal and digestive complaints, surveys from Algeria and India also report that, despite numerous plants being used, a limited number of taxa carry most of the use-reports for diarrhoea and other gut or hepato-renal disorders [122, 139]. In contrast, for chronic and highly prevalent conditions such as hypertension, type 2 diabetes, and cough, the review revealed no single dominant species; leading plants such as *Apium graveolens* and *Muntingia calabura* contributed only a small fraction of use-reports and appeared in a minority of relevant studies. Comparable patterns have been described in Mindanao, where region-specific species contribute disproportionately to local identity and may include threatened or endemic taxa requiring urgent conservation attention [29].

Taken together, this systematic review synthesises ethnobotanical data on medicinal plant use across Indonesia using a standard clinical system (ICPC-2) and appraisal of methodological quality. By harmonising 3,202 use-reports for 791 species from 73 studies, the review moves beyond simple species inventories to identify geographically widespread species, region- and ethnicity-specific plants, and priority plants for ailments. The combination of quality assessment, taxonomic scrutiny, geographical mapping and cross-ethnic analysis provides a multi-layered picture of Indonesia’s medicinal plant knowledge that is directly comparable with evidence from other parts of Asia and Africa.

At the same time, several limitations must be acknowledged when interpreting these results. First, the review is based solely on published ethnobotanical studies, which introduces publication and language bias and likely underrepresents knowledge held in grey literature, local reports or communities that have never been studied. The marked geographical skew in the underlying evidence means that some of the observed patterns such as the prominence of particular islands or species may partly reflect research effort rather than true national prevalence. Second, heterogeneity and incomplete reporting in the primary studies required substantial post-hoc standardisation. Mapping diverse emic illness concepts onto ICPC-2 categories, harmonising plant names and reconstructing quantitative indices inevitably involved interpretive decisions and may have introduced misclassification. The quality appraisal itself is constrained by what authors chose to report. Studies may have followed good ethical or taxonomic practice without documenting it, leading us to underestimate their rigour.

Finally, the cross-sectional and descriptive nature of almost all included studies limits any inference about effectiveness or safety. High use-report counts and dominance for specific plants should not be equated with proven clinical benefit. Our synthesis therefore identifies promising candidates and structural gaps rather than delivering definitive guidance for clinical practice. Future research should address these limitations by expanding ethnobotanical documentation into under-studied provinces and ethnic groups, improving taxonomic and ethical reporting, prospectively aligning data collection with international diagnostic classifications and quantitative standards, and coupling field studies with rigorous pharmacological, toxicological and, where appropriate, clinical investigations.

## Conclusion

This systematic review demonstrates that Indonesia possesses an exceptionally diverse yet unevenly documented ethnomedicinal landscape. While 791 medicinal plant species and 3,202 use-reports were synthesized, documentation was found to be concentrated in a subset of provinces, leaving significant biocultural reservoirs under-represented. Within this heterogeneous context, a culturally salient core medicinal flora shared across multiple ethnic groups was identified, distinct from highly localized species that reflect unique biocultural adaptations. Furthermore, a dual pattern of usage was observed: a small number of dominant consensus species are utilized for acute conditions, whereas chronic non-communicable diseases are managed through diverse medicinal selections. Concurrently, critical methodological gaps were revealed, particularly regarding taxonomic validation, ethical reporting, and disease standardization, which currently constrain the integration of these data into pharmacological and conservation frameworks. These findings highlight the strategic value of Indonesia’s medicinal plant knowledge and underscore the necessity for future initiatives to prioritize under-studied regions, enforce voucher-based and ethical standards, and advance pharmacological research on both high-consensus and locally unique species to ensure sustainable and evidence-informed utilization.

## Supplementary Information

Below is the link to the electronic supplementary material.

Supplementary Material 1

Supplementary Material 2

Supplementary Material 3

Supplementary Material 4

Supplementary Material 5

## Data Availability

All data generated or analysed during this study are included in this published article and its supplementary information files.

## Declarations

### AI use declarations

The authors declare that generative artificial intelligence (AI) tools were used to improve the readability, clarity, and language quality of this manuscript. Specifically, ChatGPT (OpenAI) and Nature Research Assistant were employed to assist with grammar refinement, restructuring of sentences, and enhancement of academic writing style. No AI tools were used to generate, analyse, interpret data, or create any scientific content. The authors reviewed, verified, and take full responsibility for the accuracy and integrity of all content in the manuscript.
