# Supplementary material for: A systematic review of ethnobotanical study in Indonesia: diversity and cultural patterns of medicinal plant use
Source: J Ethnobiol Ethnomed. 2026 Mar 16;22:42. doi: 10.1186/s13002-026-00879-4 (PMC13104509; doi:10.1186/s13002-026-00879-4)
Supplement: Supplementary file 1 — Supplementary Material 1 [file 13002_2026_879_MOESM1_ESM.pdf]

NIHR | National Institute for Health and Care Research

PROSPERO
International prospective register of systematic reviews

Ethnobotanical Study of Medicinal Plants in Indonesia: A Systematic Review

Raden Maya Febriyanti, Raden Bayu Indradi, Intan Timur Maisyarah, Zelika Mega Ramadhania, Ami Tjitraresmi

Citation
Raden Maya Febriyanti, Raden Bayu Indradi, Intan Timur Maisyarah, Zelika Mega Ramadhania, Ami Tjitraresmi. Ethnobotanical Study of Medicinal Plants in Indonesia: A Systematic Review. PROSPERO 2025 CRD420251136256. Available from https://www.crd.york.ac.uk/PROSPERO/view/CRD420251136256.

REVIEW TITLE AND BASIC DETAILS

Review title
Ethnobotanical Study of Medicinal Plants in Indonesia: A Systematic Review

Condition or domain being studied
We will synthesise primary ethnobotanical surveys of medicinal plant use among Indonesian ethnic groups, mapping indications to ICPC-2 and quantifying cross-cultural prominence using RFC, UV, ICF, FL, and CEPI

Rationale for the review
Indonesia's exceptional biocultural diversity is documented in many small, heterogeneous ethnobotanical studies that use non-standard ailment terms, inconsistent taxonomy, and variable reporting of informants, vouchers, and ethics. This fragmentation prevents robust national inference and limits the reuse of community knowledge for health policy, conservation, and ethnopharmacology.

This review will deliver a protocol-driven, country-wide synthesis that (i) reconciles plant names to accepted taxa; (ii) maps verbatim indications to ICPC-2 disease categories; and (iii) quantifies cultural importance using established indices—Relative Frequency of Citation (RFC), Use Value (UV), Informant Consensus Factor (ICF), and Fidelity Level (FL)—plus a cross-ethnic prominence metric (CEPI) that captures distribution across ethnic groups and island regions. Treating each study as one informant, we will generate harmonised matrices (plant × ailment/region/ethnicity), identify widely shared versus ethnicity-specific taxa, and highlight documentation gaps.

The review adds nationally comparable evidence, a transparent codebook, and an open, citable dataset to support comparative ethnopharmacology, integrative primary-care planning, and biocultural conservation

Review objectives

Primary objective

To produce an Indonesia-wide, taxonomically validated and ICPC-2–mapped synthesis of medicinal-plant uses, and to quantify cross-cultural prominence using RFC, UV, ICF, FL, and a composite CEPI metric.

Specific questions

1. What are the family/species richness and which families contribute most to the medicinal ethnoflora?

2. Which species have the highest RFC and UV overall and within ICPC-2 categories?

3. What is the ICF for each ICPC-2 category, and which species show the highest FL% per category (using pre-specified Nx thresholds)?

4. How are uses distributed across plant parts, preparations, and routes of administration?

5. Across ethnic groups, provinces, and island regions, which taxa meet the cross-ethnic prominence criteria (≥3 ethnic groups, ≥2 island regions, FC ≥2), and which are ethnicity-specific?

6. Do family richness and other availability proxies predict counts of useful species?

7. Which taxa and geographies should be prioritised for conservation and ethnopharmacological follow-up, considering prominence and harvest-sensitive parts (e.g., bark, root)?

8. Where are the major documentation gaps (vouchers, ethics/consent, UR definitions, ICPC-2 mapping, demographics), and how might they bias inference?

9. How do findings change under sensitivity analyses (alternative ailment groupings, exclusion of high-risk studies, CEPI weight variants)?

Keywords

Systematic review; Traditional medicine; Herbal medicine; Ethnobotany; Medicinal plants; Community health; Indigenous knowledge; Phytotherapy; Biodiversity conservation; Cultural diversity; Indonesia

Country

Indonesia

ELIGIBILITY CRITERIA

Population

Included

Humans; community members; Indigenous/ethnic groups; all ages/sexes; Indonesia (all island regions/provinces)

Intervention(s) or exposure(s)

Included

Use of medicinal plants; traditional/folk remedies; household/herbal therapies; ethnobotanical practices; preparations (decoction/infusion etc.)

Comparator(s) or control(s)

This review does not have any comparators

Study design

Both randomized and nonrandomized study types will be included.

Included

Observational; cross-sectional ethnobotanical surveys; qualitative/mixed-methods interviews/observation; community/primary-care settings

Context

Setting and eligibility-defining characteristics

- Geographic scope: Indonesia only (Sumatra, Java, Kalimantan/Borneo, Sulawesi, Bali–Nusa Tenggara, Maluku, Papua).
- Setting: Community/primary-care contexts (rural, peri-urban, urban); field ethnobotany with human informants in households, villages, or healer practices.
- Population: Community members, traditional healers, birth attendants, elders, caregivers; all ages/sexes. Studies based solely on literature, herbarium labels, or clinical charts without informants are excluded.
- Study designs: Primary field surveys (cross-sectional; qualitative/mixed methods) using interviews, observation, free-listing, focus groups, PRA. Laboratory-only pharmacology/phytochemistry is excluded.
- Outcomes domain: Health-related medicinal uses (therapeutic intent required). Non-medicinal uses are excluded unless a therapeutic indication is explicit.
- Minimum reporting for inclusion: Identification to ≥ genus (prefer species) with a verifiable method (voucher/herbarium acronym, expert determination, keys/databases); extractable medicinal-use data (verbatim indication and/or mappable to health categories), plus at least one of: informant counts, frequency of citation, or use-report detail enabling indices; core metadata (province/island, methods, sampling). Vouchers and ICPC-2 mapping are recorded when available but not mandatory for inclusion.
- Languages/timeframe: English or Indonesian; no date limits.
- Publication types: Peer-reviewed articles, theses, and NGO/government reports with full primary methods. Reviews/editorials/abstracts are excluded. Duplicates/supersets resolved by retaining the most complete study.
- Access: Full text must be obtainable after reasonable attempts.

TIMELINE OF THE REVIEW

Date of first submission to PROSPERO
29 August 2025

Review timeline
Start date: 29 August 2025. End date: 29 October 2025.

Date of registration in PROSPERO
29 August 2025

AVAILABILITY OF FULL PROTOCOL

Availability of full protocol
A full protocol has been written and uploaded to PROSPERO. The protocol will be made available after the review is completed.

SEARCHING AND SCREENING

Search for unpublished studies
Only published studies will be sought.

Main bibliographic databases that will be searched
The main databases to be searched are *PubMed* and *Scopus*.

Other important or specialist databases that will be searched
Garuda (Garba Rujukan Digital Indonesia)

Search language restrictions
The review will only include studies published in English and Indonesian.

Search date restrictions
There are no search date restrictions.

Other methods of identifying studies
No other methods will be used.

Additional information about identifying studies
hand-search of key journals, grey literature (institutional repositories) where methods allow extraction

Link to search strategy
A full search strategy is available in the full protocol as described in the *Availability of full protocol* section

Selection process
Studies will be screened independently by at least two people (or person/machine combination) with a process to resolve differences.

Other relevant information about searching and screening
None

DATA COLLECTION PROCESS

Data extraction from published articles and reports

Data will be extracted by one person (or a machine) and checked by at least one other person (or machine).

Authors will be asked to provide any required data not available in published reports.

Study risk of bias or quality assessment

Risk of bias will be assessed using:

JBI Critical Appraisal Checklist for Analytical Cross-Sectional Studies, adapted for ethnobotany (adds items on: definition of use-reports, taxonomic verification/voucher/herbarium reporting, and ICPC-2 mapping)

Data will be assessed by one person (or a machine) and checked by at least one other person (or machine).

Additional information will **not** be sought from study investigators if required information is unclear or unavailable in the study publications/reports.

Reporting bias assessment

Risk of bias due to missing results will not be assessed

Certainty assessment

We will assess certainty of findings using approaches aligned to the evidence type:

- Quantitative outcomes (indices/proportions): GRADE adapted for non-interventional observational data. We will judge certainty across domains risk of bias (from JBI appraisal) and indirectness (applicability of settings, taxonomy, and ICPC-2 mapping).
- Qualitative/descriptive conclusions (patterns of use, parts/preparations/routes, cross-cultural narratives where pooling isn't feasible): GRADE-CERQual with judgments on methodological limitations, coherence, adequacy of data, and relevance.

Outputs: Summary-of-Findings tables listing each key outcome (e.g., Digestive ICF, top-RFC species, category-specific FL leaders, cross-ethnic prominence) with certainty ratings and justification notes. If meta-analysis is not possible, we will provide a formal narrative certainty assessment using the same domains.

OUTCOMES TO BE ANALYSED

Main outcomes

Below are the main outcomes and how we will define, measure, and synthesise each.

1. Relative Frequency of Citation (RFC) — per species

- Definition: RFC = FC / N (range 0–1).
- Measurement/instruments: Counts from primary field studies (semi-structured interviews, free-listing, observation, focus groups).
- Time point(s): At the time of each study's data collection (cross-sectional).

- Effect measure for synthesis: Proportion with 95% CI; random-effects meta-analysis of study-specific RFC where comparable.

2. Use Value (UV) — per species

- Definition: UV = UR_total / N (range 0–∞, typically ≤1).
- Measurement/instruments: Study-level UR derivable from reported species×ailment mentions.
- Effect measure: Proportion/rate with 95% CI; random-effects pooling where comparable.

3. Informant Consensus Factor (ICF) — per ICPC-2 category

- Definition: ICF = (Nur – Nt) / (Nur – 1), where Nur = total UR in the category; Nt = number of taxa used.
- Measurement/instruments: Study-level counts mapped to ICPC-2.
- Effect measure: Continuous index (0–1); pooled (aggregate-data) ICF per category with 95% CI; study-level meta-analysis if reported consistently.

4. Fidelity Level (FL, %) — per species×ICPC-2 category

- Definition: FL% = (Np / Nx) × 100; Np = studies citing the species for that category; Nx = studies citing the species for any use.
- Measurement/instruments: Derivable from study tables; Nx thresholds (e.g., ≥3) applied in sensitivity analyses.
- Effect measure: Proportion (%) with 95% CI; random-effects pooling where comparable.

5. Cross-Ethnic Prominence Index (CEPI) — per species

- Definition: z-score sum of RFC, number of distinct ethnic groups, and number of island regions (higher = broader/culturally prominent).
- Measurement/instruments: Derived from harmonised study metadata (ethnicity, region).
- Effect measure: Standardised score and rank; threshold flag (≥3 ethnic groups, ≥2 regions, FC ≥2).

Additional outcomes

1. Distribution of uses by ailment, plant parts, preparations, routes

- Definition: Proportions of UR across ICPC-2 categories; controlled vocabularies for parts/preparations/routes.
- Measurement/instruments: Extracted from primary studies and harmonised.
- Time point(s): As above.
- Effect measure: Counts and percentages (95% CI where applicable); visualised via heatmaps.

2. Diversity and availability–use relationships

- Definition: Family/species richness; association between family richness and number of useful species.
- Measurement/instruments: Taxonomically validated species lists (POWO/WFO).
- Time point(s): As above.
- Effect measure: Counts, proportions; correlation/regression coefficients with 95% CI.

PLANNED DATA SYNTHESIS

Strategy for data synthesis

Where ≥3 comparable studies report a metric, we will pool RFC/UV (proportions) using random-effects meta-analysis, summarise ICF per ICPC-2 category, and present FL% (with Nx thresholds) and CEPI descriptively; heterogeneity (I^2/τ^2), 95% CIs/PIs, and sensitivity analyses will be reported.

CURRENT REVIEW STAGE

Stage of the review at this submission

Review stage	Started	Completed
Pilot work	✓	✓
Formal searching/study identification	✓	✓
Screening search results against inclusion criteria	✓	✓
Data extraction or receipt of IPD		
Risk of bias/quality assessment		
Data synthesis		

Review status

The review is currently planned or ongoing.

Publication of review results

Results of the review will be published in English.

REVIEW AFFILIATION, FUNDING AND PEER REVIEW

Review team members

Dr Raden Maya Febriyanti (review guarantor and contact) ORCID: 0000-0002-3437-3011. Universitas Padjadjaran. Indonesia.

No conflict of interest declared.

Dr Raden Bayu Indradi. ORCID: 0000-0002-2223-6925. Universitas Padjadjaran. Indonesia.

No conflict of interest declared.

Dr Intan Timur Maisyarah. ORCID: 0009-0001-2296-6768. Universitas Padjadjaran. Indonesia.

No conflict of interest declared.

Dr Zelika Mega Ramadhania. ORCID: 0000-0001-9872-8240. Universitas Padjadjaran. Indonesia.

No conflict of interest declared.

Dr Ami Tjitraresmi. ORCID: 0000-0002-7990-0567. Universitas Padjadjaran. Indonesia.

No conflict of interest declared.

Named contact

Dr Raden Maya Febriyanti (raden.maya@unpad.ac.id). ORCID: 0000-0002-3437-3011. Universitas Padjadjaran. Indonesia.

Review affiliation

Department of Biological Pharmacy, Faculty of Pharmacy, Universitas Padjadjaran, Indonesia

Funding source

Review has no specific/external funding but is supported by guarantor/review team (non-commercial) institutions.

Additional information about funding
This review is funded by Internal Research Grant Universitas Padjadjaran

Peer review

There has been no peer review of this planned review.

ADDITIONAL INFORMATION

Review conflict of interest

Declared individual interests are recorded under team member details.. No additional interests are recorded for this review.

Medical Subject Headings

Plants, Medicinal; Phytotherapy; Medicine, Traditional; Complementary Therapies; Indigenous Peoples; Indonesia; Cross-Sectional Studies; Surveys and Questionnaires; Qualitative Research; Systematic Reviews as Topic; Meta-Analysis as Topic; Biodiversity; Conservation of Natural Resources

SIMILAR REVIEWS

Check for similar records already in PROSPERO

PROSPERO identified a number of existing PROSPERO records that were similar to this one (last check made on 29 August 2025). These are shown below along with the reasons given by that the review team for the reviews being different and/or proceeding.

- Systematic review and meta-analysis of Traditional Medicinal Plants for Treatment of Pulmonary Tuberculosis in Africa [published 23 August 2025] [CRD420251131864]. The review was judged **not to be similar**
- Aflatoxin Contamination in Medicinal Plants Across Africa: A Systematic Review and Meta-Analysis [published 3 May 2024] [CRD42024538734]. The review was judged **not to be**

similar

- Ethnopharmacological Insights, Phytochemical Profiles and Antiviral Properties of Medicinal Plants Used Against Poxviruses in East Africa: A Systematic Review [published 4 November 2024] [CRD42024605699]. The review was judged **not to be similar**

PROSPERO version history

- Version 1.0, published 29 Aug 2025

Disclaimer

The content of this record displays the information provided by the review team. PROSPERO does not peer review registration records or endorse their content.

PROSPERO accepts and posts the information provided in good faith; responsibility for record content rests with the review team. The guarantor for this record has affirmed that the information provided is truthful and that they understand that deliberate provision of inaccurate information may be construed as scientific misconduct.

PROSPERO does not accept any liability for the content provided in this record or for its use. Readers use the information provided in this record at their own risk.

Any enquiries about the record should be referred to the named review contact
