# Supplementary material for: A systematic review of ethnobotanical study in Indonesia: diversity and cultural patterns of medicinal plant use
Source: J Ethnobiol Ethnomed. 2026 Mar 16;22:42. doi: 10.1186/s13002-026-00879-4 (PMC13104509; doi:10.1186/s13002-026-00879-4)
Supplement: Supplementary file 2 — Supplementary Material 2 [file 13002_2026_879_MOESM2_ESM.pdf]

SUPPLEMENTARY FILE S1

Summary and Characteristics of the Included Studies

Table S1. Summary of Included Studies

No.	Author	Province	Ethnic Group(s)	Sampling	Data Collection	Taxonomic Identification	# Informants	# Species Reported (general)	# Species (medicinal use)	Ethics Approval	Indices Reported
1	Nursamsu, et al. [47]	Aceh	Aneuk Jamee	Purposive Snowball	Semi-structured interviews, questionnaire	Photos & Web database	11	152	152	No formal approval	UV, RFC, FIC
2	Wiryono et al. [37]	Bengkulu	Rejang	Snowball and Convenience	Homegarden survey, visual prompts (photos)	Photos	73	130	44	No mention	None
3	Henri et al. [48]	Riau	Malays	Purposive and Snowball	Observation, semi-structured interviews	Lab/ Herbarium (no voucher numbers)	37	117	117	Permission from village heads	UV, FL, FIC, RFC
4	Rahayu,S.M., et al. [45]	West Nusa Tenggara	Local Community	Snowball	Interviews, field observation	Uni lab, standard flora books	40	87	87	No mention	Percent ages only
5	Albar, H. et al [43]	West Nusa Tenggara	Local Community	Snowball and Purposive	Semi-structured questionnaires	Mobile App & Photos	45	50	50	Permission from village heads	UV, FL, FIC
6	Sutomo, et al. [31]	South Kalimantan	Banjar	Purposive	Semi-structured questionnaire	Local names, photos	2	31	31	No formal statement	None
7	Husaini, I.P.A., et al. [49]	South Sulawesi	Local Community	Snowball	Semi-structured interviews	Web databases	60	74	74	No statement	UV, FL, FIC
8	Tamalene, M.N., et al. [39]	North Maluku	Tobelo Dalam (Togutil)	Purposive and Snowball	Semi-structured interviews, group discussions	Laboratory of Botany Bogoriense	151	93	93	Agreement obtained (ISE 2006)	Freque ncy of use
9	Husain, F. et al. [50]	Central Java	Diverse ethnicities	Purposive and Snowball	Observations, interviews, documentation	Not stated	18	38	38	No statement	None
10	Mentari, P.P., et al. [41]	West Java	Local Community	Purposive	In-depth interviews, field observations	Morphology books, online portals	30	101	101	FPIC obtained (ISE 2006)	UV, RFC, ICS
11	Suciyati, A., et al. [51]	North Kalimantan	Tidung	Snowball	Questionnaires, semi-structured interviews	No specific ID method	65	60	24	No statement	AI, FUI, PUI
12	Torimbanu, A.R., et al. [52]	Yogyakarta	Local Community	Purposive and Snowball	Questionnaire, interviews	Documentation/ interviews only	81	73	73	No statement	RFC, UV, IAR, FIC

13	Ernawita et al. [32]	Aceh	Local Community	Purposive	Open-ended questionnaire	Herbarium (for unidentified)	70	74	47	Informed consent obtained	RFC, UV, FIC
14	Tisrin M.D., et al. [53]	Riau	Akit	Purposive and Snowball	Interviews, Observation, Documentation	Field observations	NR	40	40	No statement	None
15	Afrianto W.F. [54]	East Java	Javanese	Purposive	Direct sampling, interviews	Verified via Web	55	71	17	No statement	UR, RFC, UV
16	Suwardi, A.B., et al. [55]	Aceh	Local Community	Snowball	Observation, semi-structured in-depth interviews	Herbarium Andalas Univ	120	56	16	No statement	FIC, RFC
17	Agustina, N. et al. [56]	East Java	Local Community	Purposive	Survey, interviews, recording	Online App	60	59	59	No statement	None
18	Andila, P.S., et al. [30]	Bali	Balinese	Purposive (Healers)	Interviews, questionnaires	Botanist verification	9	66	66	No statement	UV, FUV, PPV
19	Navia, Z.I et al. [57]	Aceh	Local Community	Random	Semi-structured questionnaires	Univ. Bio Lab	306	276	111	Oral prior informed consent	RFC, UV, FIC
20	Alfinandah, A.,et al. [46]	West Java	Local Community	Snowball and Purposive	Interviews, field/participant observation	Books, Online	106	48	12	Verbal consent, Village permission	RFC, UV, FIC
21	Jannaturrayyan, S., et al. [58]	West Nusa Tenggara	Local Community	Purposive and Snowball	Observation, interviews, documentation	Photos, Vouchers (preservation cited)	16	86	20	No statement	ICS
22	Mela, Y.J.A., et al. [35]	East Nusa Tenggara	Bunaq	Purposive (Shamans)	Guided field walks	Cross-check with books and database	7	63	63	No statement	SUV
23	Sutomo et al. [59]	South Kalimantan	Banjar, Javanese	Purposive (Healers)	Semi-structured interviews	Collaboration for ID	4	52	52	Informed consent obtained	None
24	Putri, L.S.E., et al. [60]	West Java	Local Community	Random	Observations, discussions, interviews	Direct field ID, Books	47	32	32	No statement	None
25	Supiandi, M.I., et al. [25]	West Kalimantan	Dayak Jangkang	Purposive and Snowball	Semi-structured interviews	Herbarium Bogoriense	10	40	40	No statement	UV, FIC, FL
26	Triyanto, A. et al. [61]	Central/ East Java	Local Community	Purposive and Snowball	Interviews	Books, Online	90	49	12	Prior informed consent	UV, RFC

27	Henri, et al. [62]	Sumatra	Local Community	Purposive and Snowball	Observation, interviews	Books	45	55	55	No statement	UV, FL, RFC
28	Santhyami et al. [63]	Central Java	Market Traders	Total sampling (Census)	Inventory, interviews	Books/ Apps	40	76	76	No statement	UV, RFC, FL
29	Jadid, N., et al. [18]	East Java	Tengger	Purposive	Semi-structured interviews	Taxonomist verification	52	30	20	Research permit	UV, FL, FIC
30	Nasution, A., et al. [64]	North Sumatra	Mandailing	Purposive	Interviews, field observations	Herbarium Bogoriense	NR	81	81	No statement	Descrip tive
31	Sari, R.Y.,et al. [65]	West Kalimantan	Dayak	Purposive	Interviews only	Unclear method	NR	51	51	No statement	Descrip tive
32	Kristianti et al. [42]	Central Kalimantan	Local Community	Purposive	Interviews only	Unclear method	NR	48	48	No statement	Descrip tive
33	Qasrin, U. et al. [66]	Riau Islands (Kepri)	Malay	Purposive/ Snowball	Interviews, observation	Unclear method	NR	53	53	No statement	Descrip tive
34	Indriati, G. [67]	Jambi	Suku Anak Dalam	Snowball	Interviews, observation	Unclear method	4	39	39	No statement	UV
35	Nurrosyidah, I.H., et al. [68]	East Java	Local Community	Purposive	Interviews only	Verified at Institution	2	55	55	Informed consent obtained	UV, FIC, FL
36	Atok, A.R.,et al. [69]	East Nusa Tenggara	Bunaq	Purposive/ Snowball	Participatory observation	Herbarium verification	25	257	69	No statement	ICS
37	Haryanti, E.S., et al. [70]	West Kalimantan	Local Community	Purposive/ Snowball	Field observation, interviews	Dendrology Lab	25	150	NS	No statement	Descrip tive
38	Royyani, M.F. and Efendy, O. [71]	North Kalimantan	Dayak	Random	Interviews, observation	Herbarium verification	NR	77	9	No statement	Descrip tive
39	Tan, A.Y.,et al.[72]	Southeast Sulawesi	Buton	Purposive	Interviews only	Books and Apps	5	41	41	No statement	Descrip tive
40	Kause, J.V.D., et al. [73]	East Nusa Tenggara	Local Community	Snowball	Interviews, observation	Books and Apps	8	20	20	No statement	Descrip tive
41	Wulandara, F.W., et al. [74]	West Kalimantan	Malay	Purposive	Interviews, observation	Unclear method	8	93	93	No statement	Descrip tive
42	Nomleni, F.T., et al. [75]	East Nusa Tenggara	Local Community	Snowball	Interviews only	Unclear method	30	31	31	No statement	Descrip tive
43	Lovadi, I., et al. [76]	West Kalimantan	Dayak Salako	Purposive/ Snowball	Interviews, observation	Herbarium verification	18	85	85	No statement	ICS
44	Kartika, T. et al. [33]	South Sumatra	Local Community	Purposive	Interviews, field observation	Field ID	NR	22	22	No statement	Descrip tive
45	Syamsiah et al. [77]	West Sulawesi	Local Community	Purposive	Interviews, observation	Unclear method	7	35	35	No statement	Descrip tive
46	Rifandi, M. et al. [78]	South Kalimantan	Local Community	Purposive	Interviews only	Unclear method	13	18	18	No statement	Descrip tive

47	Hidayat, M. and Arryati, H. [79]	Central Kalimantan	Dayak Bakumpai	Snowball	Interviews only	Field ID	5	20	20	No statement	Descrip tive
48	Mariska I., et al. [80]	South Kalimantan	Dayak Meratus	Purposive	In-depth interviews	Unclear method	3	30	30	No statement	Descrip tive
49	Pujihastuti, L.S., et al. [44]	South Sumatra / Jambi	Suku Anak Dalam	Purposive	Interviews only	Books	3	93	93	No statement	Descrip tive
50	Kiat, F.A., et al. [81]	Maluku	Local Community	Purposive	Interviews only	Books	34	71	71	No statement	Descrip tive
51	Hafizi, A., et al. [36]	South Kalimantan	Dayak Meratus	Purposive/ Snowball	Interviews only	Unclear method	2	20	20	No statement	Descrip tive
52	Daud, Y. and Manu, T.S.N. [82]	East Nusa Tenggara	Local Community	Purposive	Interviews, observation	Books	20	20	20	No statement	Descrip tive
53	Silalahi, M., et al. [16]	North Sumatra	Batak Mandailing	Purposive/ Snowball	Participatory observation, interviews	Herbarium verification	40	163	163	No statement	UV
54	Gustina et al. [83]	East Nusa Tenggara	Suku Colol	Purposive	Interviews only	Books and Herbarium	30	10	10	No statement	Descrip tive
55	Adriadi, A. et al. [84]	Jambi	Local Community	Purposive/ Snowball	Field walk, interviews	Herbarium verification	60	67	67	No statement	UV
56	Sarina, A., et al. [34]	South Sumatra	Ogan	Purposive	Observation, interviews	Herbarium verification	NR	65	65	No statement	Descrip tive
57	Nasution, J., et al. [85]	North Sumatra	Batak Toba	Purposive	Observation, interviews	Unclear method	20	40	40	No statement	Descrip tive
58	Maretta, G., et al. [86]	Lampung	Local Community	Purposive	Interviews, observation	Books	100	46	46	No statement	Descrip tive
59	Suwardi, A.B., et al. [87]	West Sumatra	Minangkabau	Random	Semi-structured interviews	Herbarium verification	393	75	13	Verbal consent	UV, RFC, CI, ICS
60	Wulandari, C., et al. [88]	Jambi	Local Community	Purposive	Interviews, field observation	Unclear method	60	66	66	No statement	ICS
61	Fathurrahman, F., et al. [89]	Central Sulawesi	Kaili Inde	Purposive	Transect walk, interviews	Herbarium Celebense (UNTAD)	NR	66	66	No statement	ICS
62	Mamahani, A.F., et al. [90]	North Sulawesi	Tonsawang	Purposive	Interviews only	Explorative survey (No vouchers)	2	40	40	No statement	Descrip tive
63	Dewantari, R. et al. [91]	Central Java	Local Community	Survey	Interviews only	Unclear method	NR	27	27	No statement	Descrip tive
64	Oktavia, G.A.E., et al. [92]	Bali	Local Community	Purposive	Observation, interviews	Bali Botanic Garden	5	69	69	No statement	Descrip tive

65	Sinaga, A.H. and Manalu, A.I. [93]	North Sumatra	Batak	Purposive	Interviews, field survey	Herbarium specimens	12	16	16	No statement	UV, RFC, RI
66	Hidayat, S., et al. [94]	West Java	Sundanese	Purposive/ Snowball	Observation, interviews, participation	Herbarium Bogoriense (LIPI)	68	150	150	No statement	UV, ICS
67	Asmemare, K. et al. [95]	Banten	Local Community	Purposive	Observation, interviews	Unclear method	88	72	32	No statement	Descrip tive
68	Tima, M.T., et al. [96]	East Nusa Tenggara	Local Community	Purposive	Interviews, field walk	Unclear method	135	54	54	No statement	Descrip tive
69	Wathan, N.,et al. [97]	South Kalimantan	Dayak Meratus	Purposive	Questionnaire	Literature/ Online tools	5	28	28	No statement	Descrip tive
70	Nurjannah et al. [40]	Aceh	Local Community	Purposive/ Snowball + Quota	Participatory Rural Appraisal (PRA)	Observation/ documentation	33	64	64	No statement	Descrip tive
71	Haris, R.N.H., et al. [98]	Southeast Sulawesi	Local Community	Purposive	Interviews, field observation	Biology Study Program, UHO	30	28	28	No statement	Descrip tive
72	Fajarwati, K. [99]	East Nusa Tenggara	Ende, Lio, Nataia, Dhawe	Purposive/ Snowball	Observation, interviews	Matching local names/photos	80	60	60	No statement	Descrip tive
73	Widiastuti, T.C.,et at. [100]	Central Java	Local Community	Purposive	Survey, field observation	Survey (no details)	50	44	44	No statement	Descrip tive
74	Buu, A.W., et al. [101]	Bali	Local Community	Random	Interviews, observation	Comparison, images, experts	5	28	28	No statement	Descrip tive
75	Tampublon, A.O., et al. [102]	South Kalimantan	Dayak Deah	Purposive	In-depth interviews	Unclear method	4	39	39	No statement	Descrip tive
76	Nurani, S. and Cahyanto, T. [103]	West Java	Sundanese	Purposive	Explorative survey, in-depth interviews	No method mentioned	15	32	32	No statement	Descrip tive
77	Ninawati et al. [38]	South Kalimantan	Balinese (Transmigrant s)	Snowball	Field observation, interviews	Unclear method	15	20	20	No statement	Descrip tive
78	Safitri, D.S., et al. [104]	East Kalimantan	Local Community	Purposive	Observation, interview, documentation	Literature/ apps	7	22	22	No statement	Descrip tive
79	Daeli, D.Y. [105]	North Sumatra	Local Community	Purposive	Interviews, observation	Unclear method	10	25	25	No statement	Descrip tive
80	Suciyati, A. and Retnaningati [106]	North Kalimantan	Local Community	Descriptive survey	Observation, interviews	Literature/ apps	NR	25	25	No statement	Descrip tive
81	Setiawan, A. et al. [107]	North Kalimantan	Dayak Lundayeh	Purposive	Interviews	Books	20	21	21	No statement	Descrip tive

82	Santosa, T.A., et al. [108]	Jambi	Tigo Luhah (Kerinci)	Purposive	Questionnaire/ Interview	Unclear method	15	45	45	No statement	Descrip tive
83	Maulidina, I. and Cahyanto, T. [109]	West Java	Local Community	Purposive	Interviews, observation	Books	30	29	29	No statement	Descrip tive
84	Selpi et al. [110]	West Kalimantan	Malay	Snowball	Interviews	Books/journals, Herbarium made	10	35	35	No statement	SUV
85	Adriadi, A., et al. [111]	Jambi	Local Community	Purposive	Interviews, observation	Observation/ interview only	19	65	65	No statement	PPV
86	Suri, L.A.,et al. [112]	South Kalimantan	Local Community	Snowball	Triangulation (Interview, obs, doc)	Specimens collected	55	76	76	No statement	Descrip tive
87	Andania, M.M., et al. [113]	West Sumatra	Minangkabau	Random/ general	Field observation, interviews	Observation/ documentation	39	42	42	No statement	Descrip tive
88	Agustin, R.D., et al. [114]	Maluku	Local Community	Probability proportional to size	Interviews	Unclear method	64	14	14	No statement	FC
89	Widodo, R.C., et al. [115]	Central Java	Local Community	Purposive	Interviews	Lab Biologi Farmasi UMP	100	49	49	Research permit mentioned	Descrip tive
90	Pangemanan, E.F.S., et al. [116]	North Sulawesi	Bolaang Mongondow	Purposive	Field observation, interviews	Literature/ apps	NR	67	67	No statement	Descrip tive
91	Jaya, S. and Suprihati [117]	Central Java	Local Community	Snowball	Interviews	Unclear method	10	40	40	No statement	RFC

Table S2. Characteristics of included ethnobotanical studies (n = 91)

Domain	Characteristic	Number of Studies
Geographic distribution	Sumatra	26 (28.6%)
	Kalimantan	22 (24.2%)
	Java	19 (20.9%)
	Nusa Tenggara	11 (12.1%)
	Sulawesi	7 (7.7%)
	Maluku	3 (3.3%)
	Bali	3 (3.3%)
Study setting	Rural	82 (90.1%)
	Mixed rural–urban	3 (3.3%)
	Peri-urban	3 (3.3%)
	Urban	3 (3.3%)
Sampling strategy (simplified)	Purposive only	45 (49.5%)
	Purposive + snowball	22 (24.2%)
	Snowball only	13 (14.3%)
	Random/probability-based	8 (8.8%)
	Census/total sampling	1 (1.1%)
	Survey (unspecified)	1 (1.1%)
	Descriptive survey	1 (1.1%)
Data collection	Interview & observation	57 (62.6%)
	Interview, observation & questionnaire	14 (15.4%)
	Interview only	10 (11.0%)
	Interview & questionnaire	7 (7.7%)
	Interview, observation & FGD	1 (1.1%)
	Interview & FGD	1 (1.1%)
	Interview, observation & PRA	1 (1.1%)
Taxonomic identification	Expert/herbarium-based	35 (38.5%)
	Literature-based	20 (22.0%)
	Database cross-check	9 (9.9%)
	Literature + database	2 (2.2%)
	Method not reported	24 (26.4%)
	Not specified	1 (1.1%)
Ethics and consent	Ethics approval/consent not reported (NR)	81 (89.0%)
	Formal ethics approval reported	9 (9.9%)
	Explicitly stated "no ethics approval"	1 (1.1%)
Use of quantitative ethnobotanical indices	None (descriptive only)	49 (53.8%)
	UV (Use Value)	15 (16.5%)
	ICF (Informant Consensus Factor)	10 (11.0%)
	RFC (Relative Frequency of Citation)	6 (6.6%)
	Other indices**	11 (12.1%)
