# Supplementary material for: A systematic review of ethnobotanical study in Indonesia: diversity and cultural patterns of medicinal plant use
Source: J Ethnobiol Ethnomed. 2026 Mar 16;22:42. doi: 10.1186/s13002-026-00879-4 (PMC13104509; doi:10.1186/s13002-026-00879-4)
Supplement: Supplementary file 5 — Supplementary Material 5 [file 13002_2026_879_MOESM5_ESM.pdf]

SUPPLEMENTARY FILE S2
QUALITY APPRAISAL OF THE INCLUDED STUDIES

The methodological landscape was dominated by non-probability sampling strategies. Purposive sampling was the most prevalent approach (49.5%), often explicitly targeting cultural specialists such as village healers (dukun), ritual experts (balian), or homegarden stewards *[30, 31]*. This reliance on purposive sampling reflects the stratified nature of ethnomedical knowledge transmission in Indonesia, where expertise is often lineage-based, gendered, or role-specific. For instance, studies in Aceh and Sumatra frequently targeted midwives and elder women [32–34], while research among minority groups like the Bunaq and Dayak focused on shamans [15, 35, 36]. Consequently, sample sizes in these specialist-focused surveys were often small (≤10 informants) [30, 31, 36], reflecting the limited pool of experts rather than methodological limitations.

To broaden the sampling frame, 24.2% of studies combined purposive methods with snowball sampling to access wider kinship networks. Snowball sampling alone was employed in 14.3% of studies, typically to trace social ties in dispersed communities [37, 38]. Probability-based methods, such as random sampling, were utilized in only 8.8% of studies, primarily in settings where knowledge was assumed to be evenly distributed, such as markets. Regarding data collection, the dominant triad consisted of semi-structured interviews supplemented by field observation. More complex, deliberative methodologies such as Focus Group Discussions (FGD) or Participatory Rural Appraisal (PRA) were rare, indicating that Indonesian ethnobotany remains largely extractive rather than participatory [39, 40].

Taxonomic authentication and ethical compliance

Taxonomic rigor varied significantly across the dataset, revealing a concerning gap in data validation. High-rigor identification such as expert verification or the deposition of voucher specimens in recognized herbaria was reported in 35 studies (38.5%). A further 22.0% of studies relied on literature-based identification, citing regional floras such as Flora of Java or pharmacognostic texts like Atlas Tumbuhan Obat [41, 42]. While nine studies (9.9%) explicitly reported cross-referencing global databases (e.g., POWO, GBIF) or using image-based tools like PlantNet [43, 44]. A significant proportion (26.4%) failed to report any taxonomic method. Furthermore, approximately 32% of studies relied on secondary sources or mobile applications without physical specimen verification.

Ethics reporting demonstrated a similar pattern of partial documentation. Despite working with Indigenous groups such as the Suku Anak Dalam, Tobelo Dalam, and Dayak [36, 39], 89.0% of studies did not explicitly state ethics approval or formal consent procedures. Only nine studies (9.9%) cited institutional review board. While several papers mentioned obtaining verbal consent or village permission [45, 46], the documentation rarely met contemporary international standards for research involving human subjects.

Application of quantitative ethnobotanical indices

The majority of the included studies (53.8%) were purely descriptive, lacking quantitative analysis of plant use. Among studies that employed quantitative metrics, Use Value (UV) was the most frequently reported index (16.5%), followed by the Informant Consensus Factor (ICF) (11.0%) and Relative Frequency of Citation (RFC) (6.6%). An additional 12.1% of studies utilized other indices, such as Fidelity Level. This heterogeneity in reporting reinforces the necessity of the standardized recalculation and harmonization undertaken in this systematic review to facilitate valid cross-regional comparisons.

The methodological quality of the 91 included studies was evaluated using 10 following questions, with a maximum possible score of 10.

Q1 : Clear inclusion criteria for participants (Y/N/Unclear/Partly)
Q2 : Study subjects and setting described in detail (Y/N/Unclear/Partly)
Q3 : Valid, reliable methods for identifying taxa (voucher/herbarium) (Y/N/Unclear/Partly)
Q4 : Standardized definitions of “use-report” and ailment categories (Y/N/Unclear/Partly)
Q5 : Appropriate sampling strategy and sample size justification (Y/N/Unclear/Partly)
Q6 : Strategies to address bias/recall (triangulation, prompts) (Y/N/Unclear/Partly)
Q7 : Adequate description of data-collection instruments (Y/N/Unclear/Partly)
Q8 : Ethical approval and informed consent reported (Y/N/Unclear/Partly)
Q9 : Clear analytic methods for indices (ICF/UV/FL/RFC) (Y/N/Unclear/Partly)
Q10 : Mapping to standard disease taxonomy (e.g., ICD/ICPC) (Y/N/Unclear/Partly)
QA total score (auto) = sum of Y = 1, Unclear/Partly = 0.5, N = 0

Compliance varied significantly across the ten quality criteria. The studies demonstrated high fidelity in descriptive elements. Notably, 93.4% of studies provided detailed descriptions of study subjects and settings and 67.0% clearly defined inclusion criteria for participants. However, significant gaps were observed in ethical reporting and standardization. Only 9.9% of studies explicitly reported obtaining ethical approval or informed consent, with the vast majority (86.8%) failing to mention these protocols. Similarly, standardization of medical terminology was largely absent. 93.4% of studies failed to map ailments to a standard disease taxonomy such as the ICD or ICPC. Furthermore, quantitative rigor was often lacking, as 92.3% of studies did not present clear analytic methods for indices such as Informant Consensus Factor (ICF) or Use Value (UV). While 51.6% of studies adequately described their data collection instruments, strategies to address bias and recall such as triangulation or prompts were absent in 56.0% of the literature. Only 24.2% of studies used valid, reliable methods for identifying taxa (e.g., vouchers/herbarium deposits), while 44.0% had no clear validation method reported.

Numbe	SR_ID	Title	First Author	Clear inclusion criteria for participants	Study subjects and setting described in detail	Valid, reliable methods for identifying taxa	Standardized definitions of “use-report” and ailment categories	Appropriate sampling strategy and sample size justification	Strategies to address bias/recall (triangulation, prompts)	Adequate description of data-collection instruments	Ethical approval and informed consent reported	Mapping to standard disease taxonomy (e.g., ICD/ICPC)	Total
**1**	**SR_EB_002**	The use of medicinal plants in the Aneuk Jamee tribe in Kota Bahagia, South Aceh District, Indonesia	Nursamsu, et al.	0.5<br><br>Selected "experts" via purposive snowball sampling, but specific criteria for "expertise" (e.g., years of practice) are not detailed.	1.0<br><br>Study area (4 villages) mapped and described. Respondent demographics (age, gender, education) provided.	0.5<br><br>Field identification using photos and a web database (POWO). No voucher specimens deposited in a herbarium.	1.0<br><br>Clear quantitative indices (UV, RFC, ICF) defined. Ailments categorized clearly.	1.0<br><br>Purposive snowball sampling (N=11)6. Justified to ensure comprehensive/safe data from experts.	0.0<br><br>Interviews only; no mention of distinct strategies (e.g., walk-in-the-woods) to verify recall accuracy.	1.0<br><br>Semi-structured interviews with a questionnaire covering specific data points.	0.0<br><br>No formal ethics board approval or written consent mentioned, though "kind friendliness" acknowledged.	1.0<br><br>Explicitly classifies diseases according to ICD-10 (2019 version).	6
**2**	**SR_EB_004**	The diversity of useful plants and botanical knowledge of the rejang tribe in Kepahiang District, Bengkulu Province, Indonesia	Wiryono et al.	1.0<br><br>Key informants selected for knowledge; 68 general villagers selected to test knowledge retention.	1.0<br><br>Location described (Kota Agung). Demographics recorded.	0.0<br><br>Species photographed15. No mention of taxonomic keys, experts, or voucher specimens.	0.5<br><br>Use categories listed (food, medicine)16, but no quantitative indices (UV/ICF) or strict ailment definitions.	0.5<br><br>Snowball for key informants; random/convenience for villagers (68 of 459). Selection method for villagers unclear.	1.0<br><br>Used visual prompts (photographs of 50 species) to test and verify respondent knowledge.	1.0<br><br>Clear procedure: survey homegardens first, then use photos to interview respondents.	0.0<br><br>No mention of informed consent or ethical approval.	0.0<br><br>Uses general terms (e.g., "Medicines," "Food"). No standard disease taxonomy.	5 5

**3**	**SR_EB_006**	Medicinal Plants for Traditional Treatment Used by the Malays in South Bangka Regency, Indonesia	Henri et al.	1.0<br><br>Purposive selection for healers (practicing medicine) and local users.	1.0<br><br>17 villages in 8 sub-districts23. Demographics (age, gender, education) detailed.	0.5<br><br>Thanks a biology lab/herbarium for identification, implying verification, but voucher numbers are not listed.	0.5<br><br>Calculates UV, FL, ICF, RFC 26. Ailment definitions are vernacular/broad (e.g., "Magic", "Wormy").	1.0<br><br>Purposive and snowball sampling. N=37 appropriate for specialized healer study.	0.0<br><br>Semi-structured interviews and questionnaires. No specific recall verification strategies.	1.0<br><br>Observation and systematic recording described.	0.0<br><br>No formal ethics or consent statement, though permission from village heads mentioned.	0.0<br><br>Diseases listed by local name/general symptom (e.g., "Bloating", "Magic"). No ICD mapping.	5
**4**	**SR_EB_007**	Ethnobotanical Study on Medicinal Plants in Sesaot Forest, Narmada, West Lombok, Indonesia	Rahayu, S.M., et al.	0.5<br><br>Snowball method used, but criteria for inclusion are not explicitly defined.	0.5<br><br>Location described. Demographics of specific respondents not detailed (only village aggregate data).	0.5<br><br>ID process in a university lab using standard flora books. No voucher specimens deposited.	0.0<br><br>Uses general disease terms. No quantitative ethnobotanical indices (UV, ICF) used; only percentages37.	0.0<br><br>Snowball method. Sample size (N) is not explicitly stated in methods or results.	0.5<br><br>Combined interviews with direct field observation.	1.0<br><br>Semi-structured interviews and field observation.	0.0<br><br>No mention of ethics or consent.	0.0<br><br>General terms used (e.g., "Cough," "Stomachache").	3

**5**	**SR_EB_ 008**	Ethnobotany of medicinal plants by the community in Langgudu Sub-district, Bima District, West Nusa Tenggara, Indonesia	Albar, H. et al	1.0<br><br>Purposive sampling for knowledgeable figures; snowball for healers/users.	1.0<br><br>3 villages described. Respondent demographics (age, gender) provided.	0.0<br><br>ID performed using a mobile app (PlantNet) and photographs. Not a scientifically valid method for primary ID.	1.0<br><br>UV, FL, ICF calculated. Diseases grouped into 7 explicit categories.	1.0<br><br>Snowball/Purposive. N=45 (15/village). Adequate for quantitative analysis.	0.0<br><br>Semi-structured questionnaires. No recall checks mentioned.	1.0<br><br>List of interview questions provided.	0.5<br><br>Permission from village heads acknowledged. Formal consent/IRB not stated.	0.0<br><br>Grouped by categories (e.g., dermatological), but specific diseases are vernacular (e.g., "Facilitates digestion"). Not ICD.	5 5
**6**	**SR_EB_ 009**	Ethnobotanical Study and Phytochemical Screening of Medicinal Plants Used by Local People in Belangian Village, South Kalimantan	Sutomo, et al.	1.0<br><br>Specific criteria listed: Banjar ethnic group, known as pananamba (healer), residing in village.	1.0<br><br>Location details (coordinates, walking distance) and respondent profiles (2 healers) provided.	0.5<br><br>Mentions herbarium preparation, but identification relied on local names and photos without citing specific taxonomists or voucher numbers.	0.0<br><br>No quantitative indices (UV/ICF). Disease terms are vernacular (e.g., "yellow fever", "bloody urine").	0.5<br><br>Purposive sampling resulted in only 2 respondents. Justified as they were the only pananamba, but N is very low.	0.0<br><br>Semi-structured questionnaire only. No triangulation mentioned.	1.0<br><br>Semi-structured questionnaire used (link provided).	0.0<br><br>No ethics committee approval or formal informed consent statement found.	0.0<br><br>Vernacular terms used; no mapping to ICD/ICPC.	4

**7**	**SR_EB_010**	Diversity and use of traditional medicinal plant species in Bantimurung-Bulusaraung National Park, Indonesia	Husaini, I.P.A., et al.	1.0<br>Definitions provided for Healer, Elderly, Herb Seller, and Local People (age 30-80).	1.0<br>Study area mapped (Minasatene Resort). Demographics (n=60) fully tabulated.	0.0<br>Identification via web databases (ThePlantList, POWO). No voucher specimens or expert verification mentioned.	1.0<br>Calculates UV, FL, ICF. Ailments grouped into 7 standardized categories (Jadid et al., 2020).	1.0<br>Snowball sampling for healers; general respondents. N=60 is adequate for this scope.	0.0<br>Semi-structured interviews only. No recall verification reported.	1.0<br>Interview topics listed (demographics, vernacular names, parts used, prep/app).	0.0<br>No ethics statement or informed consent reported.	0.5<br>Diseases grouped into standard categories (e.g., Skeletomuscular), though specific diseases remain broad.	6
**8**	**SR_EB_011**	Study of ethnobotany of medicinal plant of Tobelo Dalam (Togutil) ethnic group of Halmahera Island, Indonesia	Tamalene, M.N., et al.	0.5<br>Selection process (purposive/snowball) described, but specific inclusion criteria for general community unclear.	1.0<br>Three locations mapped. Sample sizes (Nomadic=64, Sedentary=87) and age groups detailed.	1.0<br>Unknown specimens identified at the Laboratory of Botany Bogoriense (LIPI). High reliability.	0.5<br>Frequency of use calculated. Ailment categories created to "facilitate informant," not standard taxonomy.	1.0<br>Purposive and snowball sampling. N=151 is robust for an ethnobotanical survey.	0.5<br>Used semi-structured interviews and group discussions, which helps triangulate data.	1.0<br>Direct contact, interviews, and discussions. Questions listed.	1.0<br>Explicitly states agreement obtained referring to ISE (2006) Code of Ethics.	0.0<br>Vernacular terms used (e.g., "Vitality for men", "Internal diseases").	6 5

**9**	**SR_EB_ 013**	The utilization of plants in traditional medicine and rituals of Karimunjawa Island community, Central Java, Indonesia	Husain, F. et al.	1.0<br>Targets specific groups: midwives, oil producers, village officials, diverse ethnicities.	0.5<br>Kemujan village described. 18 informants mentioned, but detailed demographic breakdown is missing.	0.0<br>No mention of voucher specimens, herbarium, or scientific identification methods.	0.0<br>Qualitative "thick description" used. No use-reports or standardized ailment categories.	0.5<br>Purposive/snowball. N=18 is small, acknowledged as a limitation for generalization.	1.0<br>Triangulation used: Direct observation of healing practices combined with interviews.	1.0<br>Observations, unstructured /semi-structured interviews, documentation on events described.	0.0<br>No ethics statement found.	0.0<br>Local terms used (e.g., "Masuk angin", "Sawan").	5
**10**	**SR_EB_ 015**	Ethnobotany of Medicinal Plants in Leuwiliang (Bogor), Indonesia	Mentari, P.P., et al.	1.0<br>Purposive sampling of "users of plant-based medicine".	1.0<br>11 villages mapped. Respondent demographics (age, gender, education, livelihood) charted.	0.5<br>ID via morphology books and online portals (IPNI, GBIF). Field observation used, but no vouchers deposited.	1.0<br>Calculates UV, RFC, ICS. Clear formulas provided. Ailments listed clearly.	0.5<br>Purposive sampling. N=30 across 11 villages is relatively small for a sub-district wide study.	0.5<br>Field observations at 30 sites used to verify plant presence mentioned in interviews.	1.0<br>In-depth interviews and field observations described clearly.	1.0<br>Prior informed consent (FPIC) obtained; adheres to ISE (2006) Code of Ethics.	0.0<br>General disease names used (e.g., "Diabetes", "Fever"). No ICD mapping.	6 5

**11**	**SR_EB_016**	Ethnobotanical study based on the five dimensions of basic life needs in Tidung Tribe of North Kalimantan, Indonesia	Suciyati, A., et al.	1.0<br><br>Criteria: Experience using plants based on Tidung philosophy (ngakan, baloy, etc.).	1.0<br><br>Tana Tidung District. N=65 informants from 22 families11. Map provided.	0.0<br><br>No specific ID method (herbarium/expert) mentioned; relies on interviews/questionnaires.	0.5<br><br>Calculates specialized indices (AI, FUI, PUI)12, but not standard medicinal use-reports.	1.0<br><br>Snowball sampling until data saturation13. N=65 is adequate.	0.0<br><br>Semi-structured interviews. No specific recall strategies.	1.0<br><br>Questionnaires and quantitative assessment instruments described.	0.0<br><br>No ethics statement found.	0.0<br><br>Uses local cultural categories (Sihat, Ngakan).	4	5
**12**	**SR_EB_017**	Ethnobotany of medicinal plants used by the Javanese community of Mount Merapi National Park, Central Java, Indonesia	Torimbanu, A.R., et al.	1.0<br><br>Residents of 3 specific villages. Demographics fully detailed (N=81).	1.0<br><br>3 villages mapped. Respondent demographics (gender, age, education) tabulated.	0.0<br><br>Documentation and interviews only. No herbarium/voucher mentioned.	1.0<br><br>Calculates RFC, UV, IAR, ICF. Diseases grouped into standard ICD-like categories (e.g., CID, CSD)9.	1.0<br><br>Purposive (N=81) and Snowball (N=4). Robust sample size.	0.5<br><br>Key informant interviews used to guide/validate the broader questionnaire10.	1.0<br><br>Questionnaire content detailed (habitat, habitus, part used, preparation).	0.0<br><br>No ethics statement found.	1.0<br><br>Explicitly maps diseases to categories like "Certain infectious and parasitic diseases" (ICD-10 style) [Table 3].	6	5

**13**	**SR_EB_018**	Investigation on underutilized plants used as daily medication, fruit source and traditional ceremonies in Bireuen regency, Aceh, Indonesia	Ernawita et al.	1.0<br><br>Criteria: Age >60, resident >10 yrs, good knowledge3.	1.0<br><br>17 districts. N=70. Map provided.	0.5<br><br>Unidentified samples sent to Herbarium for ID4, but voucher numbers not listed for all.	1.0<br><br>Calculates RFC, UV, ICF. Categories: Medicine, Food, Ceremony.	1.0<br><br>Purposive sampling N=70 across 17 districts.	0.5<br><br>Open-ended questionnaire allowed broad input; verification by residents mentioned.	1.0<br><br>Open-ended questionnaire covering medicine, food, and ceremony.	1.0<br><br>Informed consent obtained5.	0.0<br><br>Ailments listed (e.g., "Asthma", "Ringworm") but not mapped to ICD chapters.	7 5
**14**	**SR_EB_019**	Ethnobotany of traditional medicine in Akit Tribe, Teluk Setimbul Village, Karimun District, Indonesia	Tisrin M.D., et al.	1.0<br><br>Criteria: Customary leader, shaman, and knowledgeable individuals.	0.5<br><br>Location described. Critical: Sample size (N) of respondents is not explicitly stated in text or tables.	0.0<br><br>Field observations used to verify7, but no scientific ID method or vouchers cited.	0.0<br><br>Qualitative descriptive. No quantitative indices (UV, ICF) or standard ailment definitions.	0.0<br><br>Purposive/Snowball. Lack of N makes it impossible to judge adequacy.	1.0<br><br>Triangulation used: Interviews, Participant Observation, and Documentation8.	1.0<br><br>Interview guides and observation sheets used.	0.0<br><br>No ethics statement found.	0.0<br><br>General terms used.	3 5

**15**	**SR_EB_ 020**	Ethnobotanical documentation of home gardens in relation to Javanese basic life needs in Kediri District, East Java, Indonesia	Afrianto W.F	1.0<br><br>Criteria: Garden owners, mostly women, 40-60 years old1.	1.0<br><br>Datengan Village mapped. Sample size N=55 home gardens/owners described.	0.5<br><br>Direct sampling; IDs verified via POWO. No voucher specimens or herbarium deposit mentioned.	0.5<br><br>Calculates UR, RFC, UV, etc.2. Uses Javanese cultural categories (pangan, sandang) rather than standard medical taxonomy.	1.0<br><br>Purposive sampling based on garden criteria. N=55 is reasonable for specific village study.	0.0<br><br>Direct sampling and interviews only. No specific recall strategies noted.	0.5<br><br>Mentions "direct sampling" and obtaining info from respondents, but instrument details are sparse.	0.0<br><br>No ethics statement found.	0.0<br><br>Uses cultural categories (Loro/Health) without standard disease mapping.	4 5
**16**	**SR_EB_ 021**	Ethnobotany and conservation of indigenous edible fruit plants in South Aceh, Indonesia	Suwardi, A.B., et al.	1.0<br><br>Snowball sampling for diverse backgrounds (sex, age, etc.).	1.0<br><br>6 villages in South Aceh. N=120. Demographics detailed in Table 1.	1.0<br><br>Vouchers identified at Herbarium of Andalas University. Verified via The Plant List/IPNI.	1.0<br><br>Calculates ICF, RFC. Categories defined (food, medicine, construction, etc.).	1.0<br><br>Snowball sampling N=120 (20/village). Robust size.	1.0<br><br>Participatory observation + semi-structured in-depth interviews (triangulation).	1.0<br><br>Interview guide based on Alexiades & Sheldon (1996). Specific data points listed.	0.0<br><br>No ethics statement found.	0.0<br><br>General disease terms used.	7

**17**	**SR_EB_022**	Diversity of the medicinal plant in homegarden of local communities in the coastal area of Prigi Bay, Trenggalek, East Java, Indonesia	Agustina, N. et al.	1.0<br><br>Residents with homegardens preferred.	1.0<br><br>3 villages described. N=60. Demographics detailed.	0.0<br><br>Identification using an online app (PlantNet). Not a scientifically valid method for primary ID without expert verification.	0.0<br><br>Descriptive stats only (%). No quantitative indices or standard ailment definitions.	1.0<br><br>Purposive sampling N=60. Adequate for descriptive homegarden study.	0.5<br><br>Direct survey + interviews + recording conversations (allows verification).	1.0<br><br>Semi-structured interviews. Data points listed.	0.0<br><br>No ethics statement found.	0.0<br><br>General terms used.	4 5
**18**	**SR_EB_023**	Medicinal Plants Diversity Used by Balinese in Buleleng Regency, Bali	Andila, P.S., et al.	1.0<br><br>Explicitly targeted Balians (traditional healers).	0.5<br><br>Villages listed, but demographics only summarized for 8 informants. N=9 is very small for quantitative analysis.	1.0<br><br>Vouchers identified by taxonomists at "Eka Karya" Botanic Gardens. Highly reliable.	0.5<br><br>Calculates UV, FUV, PPV. Ailments listed by local terms (e.g., "Black magic", "Cramps") without standard coding.	0.5<br><br>Purposive sampling of 9 healers. Appropriate for specialist knowledge but limits statistical generalization.	0.0<br><br>Direct interviews and questionnaires. No triangulation mentioned.	1.0<br><br>Semi-open questionnaire covering specific data points (species, habitat, part use, etc.).	0.0<br><br>No ethics statement found.	0.0<br><br>Uses vernacular disease terms.	4 5

**19**	**SR_EB_025**	Diversity and ethnobotany of useful plants in Bandar Pusaka, Aceh Tamiang District, Indonesia	Navia, Z.I et al.	1.0<br><br>Random selection of residents ≥15 years old.	1.0<br><br>10 villages described. N=306. Demographics detailed in Table 1.	0.5<br><br>Specimens identified at Univ. Samudra bio lab. Updated via POWO. No voucher numbers listed.	1.0<br><br>Calculates RFC, UV, ICF. Use categories clearly defined.	1.0<br><br>Random sampling. Sample size calculated using Cochran formula (N=306). Robust.	0.0<br><br>Semi-structured questionnaires only. No recall verification reported.	1.0<br><br>Questionnaire topics listed.	1.0<br><br>Oral prior informed consent obtained.	0.0<br><br>General disease categories used.	6 5
**20**	**SR_EB_02**	Ethnobotany of wild edible plants by the community of Cijambu Village, Sumedang District, West Java, Indonesia	Alfinanda	1.0<br><br>Snowball sampling targeting specific roles (village head, shamans, farmers). N=20 informants + 86 general respondents.	1.0<br><br>Cijambu Village mapped. Population and land use detailed. Respondent profiles (gender, age, edu) provided.	0.0<br><br>Specimens collected but identification cited "identification books" and online databases. No voucher numbers or herbarium deposit mentioned.	1.0<br><br>Calculates RFC, UV, ICF. Uses specific disease categories (e.g., "Digestive", "Respiratory") and food categories.	1.0<br><br>Snowball + Purposive. Sample size calculated using Lynch formula (N=86). Robust.	1.0<br><br>Triangulation used: Semi-structured interviews, field observation, and participant observation.	1.0<br><br>Detailed description of interview guides and questionnaires.	0.5<br><br>Permission from village head and verbal consent mentioned. No formal ethics board cited.	0.5<br><br>Diseases grouped into categories (e.g., "Certain infectious and parasitic"), similar to ICD but not explicitly coded.	7

**21**	**SR_EB_ 028**	Ethnobotanical Study on Plants Used by Local People in Dusun Beleq, Gumantar Village, North Lombok Regency	Jannaturrayyan, S., et al.	0.5<br><br>Purposive selection of locals with knowledge. Specific criteria not fully detailed.	0.5<br><br>Dusun Beleq described. N=16 informants mentioned in results, but demographic table is missing/not cited.	0.5<br><br>Photos taken/vouchers preserved (cited methods). No specific herbarium deposit location mentioned in text.	1.0<br><br>Calculates Index of Cultural Significance (ICS). Categories defined (Food, Medicine, Ritual).	0.0<br><br>Purposive/Snowball. N=16 is quite small for a general ethnobotanical survey; justification is weak.	0.5<br><br>Observation, interviews, and documentation used.	0.5<br><br>Mentions observation and interviews. No specific instrument details.	0.0<br><br>No ethics statement found.	0.0<br><br>General terms used.	3
**22**	**SR_EB_ 029**	Ethnobotany of semi-arid medicinal plants used by Bunaq Tribe in Lamaknen, Belu District, East Nusa Tenggara, Indonesia	Mela, Y.J.A., et al.	1.0<br><br>Purposive selection of "village shamans" based on specific criteria (cured diseases, experience).	1.0<br><br>Lamaknen sub-district mapped. Key informants (N=7 shamans) described by gender.	0.5<br><br>Field ID cross-checked with Flora of Java and online databases. No voucher numbers listed.	1.0<br><br>Calculates Species Use Value (SUV). Disease categories listed in Table 1.	1.0<br><br>Purposive. N=7 is appropriate for a specialist healer study.	0.5<br><br>Guided field walks used to verify plant identification in situ.	1.0<br><br>Questionnaire topics listed (names, parts, preparation).	0.0<br><br>No ethics statement found.	0.0<br><br>General terms used (e.g., "Vomiting", "Bone fracture").	5

**23**	**SR_EB_030**	Ethnobotanical Study of Medicinal Plants of Banjar and Java Tribes in Pandansari Village, South Kalimantan	Sutomo et al.	1.0<br>Specific criteria: Healers (pananamba), Banjar/Javanese ethnicity, resident.	1.0<br>Pandansari Village. Informant profiles (N=4) detailed in Table II.	0.0<br>Two plants unidentified initially; collaboration for ID mentioned but no vouchers for the main list cited.	0.0<br>No quantitative indices (UV/ICF). General disease terms used.	0.5<br>Purposive. N=4 is very small, though targeted at experts.	0.0<br>Semi-structured interviews only.	1.0<br>Interview guide topics listed. Questionnaire link provided.	1.0<br>Informed consent obtained from all participants.	0.0<br>General terms used.	4 5
**24**	**SR_EB_031**	Ethnobotanical study of herbal medicine in Ranggawulung Urban Forest, Subang District, West Java, Indonesia	Putri, L.S.E., et al.	0.5<br>Random selection of residents aged 31-75. "Healers" mentioned but selection criteria unclear.	1.0<br>RUF area mapped. N=47 (28 female, 19 male).	0.5<br>Direct field ID. Unknowns ID'd via books. "Collection... was avoided to conserve," implies no vouchers.	1.0<br>Ailments grouped into specific categories (e.g., SMSD, ED) which seem standard-ish but not ICD.	1.0<br>Random selection. N=47 is reasonable for a preliminary survey.	0.5<br>Field observations and discussions used alongside interviews.	0.5<br>Mentions "deep personal interview and questionnaire," but instrument details are sparse.	0.0<br>No ethics statement found.	0.5<br>Uses biomedical terms (e.g., "Skeleton-Muscular System Disorder") but not full ICD coding.	5

**25**	**SR_EB_032**	Ethnobotany of traditional medicine in Dayak Jangkang Tribe, Sanggau District, West Kalimantan, Indonesia	Supiandi, M.I., et al.	1.0<br><br>Targeted Manang (shamans) and elders. Specific criteria: native, knowledgeable, >35 years old.	1.0<br><br>Jangkang sub-district (2 villages). Respondent profiles (age, gender, occupation) tabulated.	1.0<br><br>Identification verified at Herbarium Bogoriense (LIPI). Voucher specimens prepared.	1.0<br><br>Calculates UV, ICF, FL. Ailment categories are standardized (e.g., "Gastrointestinal", "Dermatological").	1.0<br><br>Purposive sampling (8 key informants) followed by snowball (12 general). N=20 is reasonable for specialized shaman knowledge.	0.0<br><br>Semi-structured interviews only. No explicit triangulation or recall strategies mentioned.	1.0<br><br>Instruments described: Observation sheets, interview guides, documentation tools.	0.0<br><br>No ethics statement found.	0.0<br><br>Grouped into broad categories, but specific diseases are vernacular (e.g., "Muntah darah").	**6**
**26**	**SR_EB_036**	Ethnobotany of wild edible plants used by local communities in three districts along the upper Bengawan Solo River, Central Java, Indonesia	Triyanto, A. et al.	0.5<br><br>Criteria: "Local community". Specific selection criteria not detailed.	1.0<br><br>3 districts mapped. N=112. Demographics (age, gender, occupation) tabulated.	0.5<br><br>Identification via books (Flora of Java) and online (POWO). No vouchers deposited.	1.0<br><br>Calculates UV, RFC. Focus is food ("Wild Edible Plants"), categories are clear (vegetable, fruit, etc.).	1.0<br><br>Purposive and Snowball sampling. N=112 is robust for general survey.	0.0<br><br>Interviews only. No triangulation mentioned.	1.0<br><br>Questionnaire items described (local name, usage, part used).	1.0<br><br>Prior informed consent mentioned.	0.0<br><br>Focus is food, but medicinal uses mentioned are general (e.g., "Diabetes").	**6 5**

**27**	**SR_EB_037**	Ethnobotanical Study of Early Childhood Medicinal Plants Used by the Local People in South Bangka Regency, Indonesia	Henri, et al.	1.0<br><br>Specific criteria: Traditional healers (dukun kampuong) and parents with young children.	1.0<br><br>8 sub-districts (17 villages). Demographics fully detailed.	0.5<br><br>Identification via books. No voucher specimens mentioned.	1.0<br><br>Calculates UV, FL, RFC. Ailments specific to early childhood.	1.0<br><br>Purposive/Snowball. N=37 (33 healers). Appropriate for specialized topic.	0.5<br><br>Direct field observation and interviews.	1.0<br><br>Semi-structured interview guide and questionnaire described.	0.0<br><br>No ethics statement found.	0.0<br><br>General disease terms (e.g., "Wormy", "Fever").	**6**
**28**	**SR_EB_038**	Exploring Urban Ethnobotany: A Case Study of Medicinal Plants Traded in Gede Hardjonagoro Market, Surakarta, Indonesia	Santhyami et al.	1.0<br><br>Criteria: Market traders of jamu ingredients.	1.0<br><br>Pasar Gede market, Surakarta. N=16 traders. Demographics detailed.	0.0<br><br>Identification via books/apps. Market samples can be hard to ID; no expert verification cited.	1.0<br><br>Calculates UV, RFC, FL. Ailments grouped by body systems.	1.0<br><br>Total sampling (census) of all 16 traders in the specific market zone.	0.5<br><br>Inventory of market stalls + interviews (triangulation of sale vs. report).	1.0<br><br>Interview guidelines and inventory sheets described.	0.0<br><br>No ethics statement found.	0.5<br><br>Ailments grouped by system (e.g., "Digestive", "Respiratory"), close to standard.	**6**
**29**	**SR_EB_039**	An ethnobotanical study of medicinal plants used by the Tengger tribe in Ngadisari village, Indonesia	Jadid, N., et al.	1.0<br><br>Targeted traditional healers (dukuns) and elders based on community recognition.	1.0<br><br>Ngadisari village mapped. Informant demographics (age, gender, education) detailed.	1.0<br><br>Voucher specimens collected and identified by taxonomist at Purwodadi Botanic Garden.	1.0<br><br>Calculates UV, FL, ICF. Ailments grouped into categories (e.g., Gastrointestinal).	0.5<br><br>Purposive sampling (N=48). Sample size justification based on "key informant" availability is acceptable.	0.0<br><br>Semi-structured interviews. No explicit triangulation mentioned.	1.0<br><br>Questionnaire topics listed (local name, part used, processing).	1.0<br><br>Research permit obtained from local authorities (Bakesbangpol, TNBTS).	0.5<br><br>Grouped into body systems, approaching standard taxonomy.	7

**30**	**SR_EB_ 040**	Ethnobotany of Mandailing Tribe in Batang Gadis National Park	Nasution, A., et al.	0.5<br>Selection criteria implied (knowledgeable locals) but not explicitly detailed.	1.0<br>Batang Gadis National Park. Demographics provided.	1.0<br>Specimens identified at Herbarium Bogoriense.	0.5<br>Descriptive stats mostly. Mentions categories but less rigorous than UV/ICF papers.	0.5<br>Purposive sampling. Sample size sufficiency not discussed.	0.0<br>Interviews and field observations.	0.5<br>General description of interview methods.	0.0<br>No ethics statement found.	0.0<br>General terms used.	3 5
**31**	**SR_EB_ 041**	Ethnobotany of Medicinal Plants in Serambai Village, Kembayan Sub-District, Sanggau, West Kalimantan	Sari, R.Y.,et al.	0.5<br>Targeted battra (healers) and community leaders.	1.0<br>Serambai Village described. Demographics detailed.	0.5<br>Identification mentioned but no specific herbarium or voucher numbers cited.	0.0<br>Qualitative/ Descriptive only (percentages).	0.5<br>Purposive sampling. N is small, justification weak.	0.0<br>Interviews only.	0.5<br>General interview guide.	0.0<br>No ethics statement found.	0.0<br>Vernacular disease terms used.	3
**32**	**SR_EB_ 042**	Studi Etnobotani Tumbuhan Obat di Desa Bani Amas, Kec. Bengkayang, Kab. Bengkayang	Kristianti et al.	0.5<br>Targeted "community members" with knowledge.	1.0<br>Bani Amas Village described. Demographics detailed.	0.0<br>Identification method unclear/not rigorous. No vouchers.	0.0<br>Qualitative/ Descriptive only.	0.5<br>Purposive sampling. Small sample size.	0.0<br>Interviews only.	0.5<br>General interview guide.	0.0<br>No ethics statement found.	0.0<br>Vernacular disease terms used.	2 5

**33**	**SR_EB_043**	Studi Etnobotani Tumbuhan Berkhasiat Obat yang Dimanfaatkan Masyarakat Suku Melayu Kabupaten Lingga Kepulauan Riau	Qasrin, U. et al.	0.5<br><br>Targeted Malay tribe members.	1.0<br><br>Lingga District described. Demographics provided.	0.0<br><br>Identification method unclear/not rigorous. No vouchers.	0.0<br><br>Qualitative/ Descriptive only.	0.5<br><br>Purposive/Snowball sampling.	0.0<br><br>Interviews and observation.	0.5<br><br>General interview guide.	0.0<br><br>No ethics statement found.	0.0<br><br>Vernacular disease terms used.	2 5
**34**	**SR_EB_044**	Etnobotani Tumbuhan Obat yang Digunakan Suku Anak Dalam di Desa Tabun Kecamatan VII Koto Kabupaten Tebo Jambi	Indriati, G.	1.0<br><br>Specific criteria: native Suku Anak Dalam, know medicinal plants, adults.	1.0<br><br>Tabun Village described. Demographics detailed (15 informants).	0.5<br><br>Identification mentioned but no specific herbarium or voucher numbers listed.	0.5<br><br>Use-value mentioned but calculations /categories not standard.	0.5<br><br>Snowball sampling. N=15. Small sample size.	0.0<br><br>Interviews and observation only.	0.5<br><br>Methods described generally.	0.0<br><br>No ethics statement found.	0.0<br><br>General terms used.	**4**
**35**	**SR_EB_045**	Studi Etnobotani Tumbuhan Obat Berbasis Pengetahuan Lokal di Desa Seloliman, Kecamatan Trawas, Kabupaten Mojokerto, Jawa Timur	Nurrosyidah, I.H., et al.	1.0<br><br>Targeted community leaders, healers (dukun), and elders.	1.0<br><br>Seloliman Village described. Demographics (N=30) tabulated.	1.0<br><br>Identification verified at Materia Medica Batu.	1.0<br><br>Calculates UV, ICF, FL. Categories clearly defined.	1.0<br><br>Purposive sampling. N=30. Adequate for focused ethnobotany.	0.0<br><br>Interviews only.	1.0<br><br>Questionnaire sheet described.	1.0<br><br>Informed consent obtained.	0.0<br><br>General disease terms used.	**6**

**36**	**SR_EB_ 046**	Etnobotani Masyarakat Suku Bunaq (Studi Kasus di Desa Dirun, Belu, NTT)	Atok, A.R.,et al.	1.0<br><br>Key informants (traditional leaders, healers) and general respondents.	1.0<br><br>Dirun Village described. Demographics (N=43) detailed.	1.0<br><br>Voucher specimens identified at Herbarium Bogoriense (LIPI).	1.0<br><br>Calculates ICS. Use categories defined.	1.0<br><br>Purposive/Snowball. N=43. Reasonable sample size.	0.5<br><br>Participatory observation used for verification.	0.5<br><br>Methods described generally.	0.0<br><br>No ethics statement found.	0.0<br><br>General terms used.	**6**
**37**	**SR_EB_ 047**	Etnobotani tumbuhan berguna oleh masyarakat sekitar kawasan KPH Model Kapuas Hulu (Studi Kasus Desa Tamao Kecamatan Embaloh Hulu Kalimantan Barat)	Haryanti, E.S., et al.	1.0<br><br>Criteria: Community figures, village officials, healers.	1.0<br><br>Tamao Village described. Demographics (N=30) detailed.	0.5<br><br>Identification mentioned (Dendrology lab), but no voucher numbers.	0.0<br><br>Descriptive only. No quantitative indices.	1.0<br><br>Purposive/Snowball. N=30. Adequate for descriptive study.	0.5<br><br>Field observation used to verify plant existence.	0.5<br><br>General interview guide.	0.0<br><br>No ethics statement found.	0.0<br><br>General terms used.	**4**
**38**	**SR_EB_ 048**	Kajian Etnobotani Masyarakat Dayak di Desa Tau Lumbis, Kabupaten Nunukan, Propinsi Kalimantan Utara, Indonesia	Royyani, M.F. and Efendy, O.	0.5<br><br>Selection criteria implied but not explicitly detailed.	1.0<br><br>Tau Lumbis Village described. Demographics (N=20) tabulated.	1.0<br><br>Vouchers identified at Herbarium Bogoriense (LIPI).	0.5<br><br>Descriptive mostly. Mentions categories but less rigorous than UV/ICF papers.	0.5<br><br>Random sampling. N=20. Small sample size.	0.0<br><br>Interviews and observation.	0.5<br><br>General interview description.	0.0<br><br>No ethics statement found.	0.0<br><br>General terms used.	**3 5**

**39**	**SR_EB_ 049**	Etnobotani Tumbuhan Obat Masyarakat Etnis Buton di Kota Baubau, Provinsi Sulawesi Tenggara	Tan, A.Y.,et al.	0.5<br>Criteria mentioned (ethnic Buton, adults, knowledgeable), but specific selection process not detailed.	1.0<br>Baubau City. Respondent characteristics (age, gender, occupation) detailed.	0.5<br>Identification via books and apps. No voucher specimens or herbarium mentioned.	0.0<br>Qualitative descriptive only.	0.5<br>Purposive sampling. N=20. Justification for sample size is weak.	0.0<br>Interviews only.	0.5<br>General interview description.	0.0<br>No ethics statement found.	0.0<br>Vernacular disease terms used.	3
**40**	**SR_EB_ 052**	Etnobotani Tumbuhan Obat di Desa Barene Kecamatan Malaka Tengah Kabupaten Malaka	Kause, J.V.D., et al.	0.5<br>Targeted "community members" with knowledge.	1.0<br>Barene Village described. Demographics (N=20) tabulated.	0.5<br>Identification via books/apps. No vouchers.	0.0<br>Qualitative/ Descriptive only.	0.5<br>Snowball sampling. N=20. Small sample size.	0.0<br>Interviews and observation.	1.0<br>Interview guide questions listed.	0.0<br>No ethics statement found.	0.0<br>Vernacular disease terms used.	3 5
**41**	**SR_EB_ 056**	Etnobotani Tumbuhan Obat Suku Melayu Desa Durian Sebatang Kecamatan Seponti Kabupaten Kayong Utara	Wulandara, F.W., et al.	1.0<br>Specific criteria: healers (dukun beranak, tukang urut) and elders.	1.0<br>Durian Sebatang Village. Respondent profiles (N=8) detailed.	0.0<br>Identification method unclear. No vouchers.	0.0<br>Descriptive only.	0.5<br>Purposive sampling. N=8. Very small sample size, justification weak.	0.0<br>Interviews and observation.	0.5<br>General interview guide.	0.0<br>No ethics statement found.	0.0<br>Vernacular disease terms used.	3

**42**	**SR_EB_057**	Etnobotani Tumbuhan Obat Tradisional di Desa Huilelot dan Desa Uiasa Kecamatan Semau Kabupaten Kupang	Nomleni, F.T., et al.	0.5<br><br>Targeted "community members" with knowledge.	1.0<br><br>Huilelot and Uiasa Villages described. Demographics (N=25) tabulated.	0.0<br><br>Identification method unclear. No vouchers.	0.0<br><br>Qualitative/Descriptive only.	0.5<br><br>Snowball sampling. N=25. Small sample size.	0.0<br><br>Interviews only.	0.5<br><br>General interview guide.	0.0<br><br>No ethics statement found.	0.0<br><br>Vernacular disease terms used.	2 5
**43**	**SR_EB_059**	Survey Etnobotani Tumbuhan Obat pada Masyarakat Dayak Salako di sekitar Cagar Alam Raya Pasi Provinsi Kalimantan Barat	Lovadi, I., et al.	1.0<br><br>Targeted battra (healers) and elders. Specific criteria listed.	1.0<br><br>Cagar Alam Raya Pasi area. Demographics (N=17) detailed.	1.0<br><br>Voucher specimens identified at Herbarium Biology Untan.	1.0<br><br>Calculates ICS. Categories defined.	0.5<br><br>Purposive/Snowball. N=17. Small sample size.	0.0<br><br>Interviews and observation.	0.5<br><br>General interview description.	0.0<br><br>No ethics statement found.	0.0<br><br>Vernacular disease terms used.	5
**44**	**SR_EB_062**	Studi Etnobotani Tumbuhan Obat di Desa Perajen, Banyuasin	Kartika, T. et al.	0.5<br><br>Criteria mentioned ("know and use") but vague.	1.0<br><br>Perajen Village described. Demographics (N=20) tabulated.	0.5<br><br>Field identification mentioned. No voucher specimens.	0.0<br><br>Descriptive only. No quantitative indices.	0.5<br><br>Purposive sampling. N=20. Small sample size, weak justification.	0.5<br><br>Interviews and direct field observation.	0.0<br><br>Instrument mentioned but details sparse.	0.0<br><br>No ethics statement found.	0.0<br><br>Vernacular disease terms used.	3

**45**	**SR_EB_ 068**	Kajian Etnobotani dalam Pemanfaatan Tumbuhan Obat Tradisional di Kecamatan Pana Kabupaten Mamasa, Sulawesi Barat	Syamsiah et al.	0.5<br>Criteria implied (knowledgeable people).	1.0<br>Pana District described. Demographics (N=7) detailed.	0.0<br>Identification method unclear. No vouchers.	0.0<br>Qualitative/ Descriptive only.	0.5<br>Purposive sampling. N=7 is very small.	0.5<br>Interviews and observation.	0.5<br>General interview description.	0.0<br>No ethics statement found.	0.0<br>Vernacular disease terms used.	3
**46**	**SR_EB_ 070**	Kajian Etnobotani Tumbuhan Obat Masyarakat Desa Muara Pagatan Kecamatan Kusan Hilir Kabupaten Tanah Bumbu	Rifandi, M. et al.	1.0<br>Specific criteria: elders, dukun kampung, >40 years old, experience.	1.0<br>Muara Pagatan Village described. Demographics (N=5) detailed.	0.0<br>Identification method unclear. No vouchers.	0.0<br>Descriptive only.	0.5<br>Purposive sampling. N=5 key informants. Very small.	0.0<br>Interviews only.	1.0<br>Questionnaire described.	0.0<br>No ethics statement found.	0.0<br>Vernacular disease terms used.	3 5
**47**	**SR_EB_ 071**	Etnobotani Tanaman Obat Masyarakat Suku Dayak Bakumpai Di Desa Lemo II Kec. Teweh Tengah Kab. Barito Utara	Hidayat, M. and Arryati, H.	1.0<br>Specific criteria: traditional leaders, dukun kampung, users.	1.0<br>Lemo II Village described. Demographics (N=5) detailed.	0.5<br>Field identification mentioned. No vouchers.	0.0<br>Descriptive only.	0.5<br>Snowball sampling. N=5 key respondents. Very small.	0.0<br>Interviews only.	0.5<br>General interview description.	0.0<br>No ethics statement found.	0.0<br>Vernacular disease terms used.	3 5

**48**	**SR_EB_ 072**	Etnobotani Tumbuhan Obat oleh Masyarakat Dayak Meratus Desa Haruyan Dayak, Kec. Hantakan, Kab. Hulu Sungai Tengah, Kalsel	Mariska I., et al.	1.0<br>Specific criteria: Balian (healers), elders, aparat desa.	1.0<br>Haruyan Dayak Village described. Demographics (N=5) detailed.	0.0<br>Identification method unclear. No vouchers.	0.0<br>Descriptive only.	0.5<br>Purposive sampling. N=5 key informants. Very small.	0.0<br>In-depth interviews only.	0.5<br>Questionnaire used to "focus interview".	0.0<br>No ethics statement found.	0.0<br>Vernacular disease terms used.	3
**49**	**SR_EB_ 073**	Studi Etnobotani Tumbuhan Obat Suku Anak Dalam di Desa Sungai Jernih, Kec. Rupit Kab. Musi Rawas Utara, Sumatera Selatan	Pujihastuti, L.S., et al.	0.5<br>Targeted "traditional healers" (dukun), implied but not explicitly criteria-listed.	1.0<br>Sungai Jernih Village described. Demographics (N=3) tabulated.	0.5<br>Identified via books. No voucher specimens mentioned.	0.0<br>Descriptive only.	0.5<br>Purposive sampling. N=3 healers. Very small sample size.	0.0<br>Interviews only.	0.5<br>General interview description.	0.0<br>No ethics statement found.	0.0<br>Vernacular disease terms used.	3
**50**	**SR_EB_ 074**	Etnobotani Tumbuhan Obat Tradisional di Desa Piliana dan Desa Hatu, Kec. Tehoru, Kab. Maluku Tengah	Kiat, F.A., et al.	1.0<br>Targeted specific roles (village head, customary leader, healer, farmer).	1.0<br>Piliana and Hatu Villages described. Demographics (N=20) detailed.	0.5<br>Identification via books. No vouchers mentioned.	0.0<br>Descriptive only.	0.5<br>Purposive sampling. N=20. Small sample size.	0.0<br>Interviews only.	0.5<br>General interview description.	0.0<br>No ethics statement found.	0.0<br>Vernacular disease terms used.	3 5

**51**	**SR_EB_075**	Etnobotani Tanaman Obat Masyarakat Dayak Meratus, Halong (Balangan)	Hafizi, A., et al.	1.0<br><br>Specific criteria: Batra (healers) and elders.	1.0<br><br>Halong District described. Demographics (N=10) detailed.	0.0<br><br>Identification method unclear. No vouchers.	0.0<br><br>Descriptive only.	0.5<br><br>Purposive/Snowball. N=10. Very small sample size.	0.0<br><br>Interviews only.	0.5<br><br>General interview description.	0.0<br><br>No ethics statement found.	0.0<br><br>Vernacular disease terms used.	3
**52**	**SR_EB_076**	Etnobotani Tumbuhan Obat oleh Masyarakat Desa Pukuafu, Kec. Landu Leko, Kab. Rote Ndao	Daud, Y. and Manu, T.S.N.	0.5<br><br>Targeted "community members" with knowledge.	1.0<br><br>Pukuafu Village described. Demographics (N=20) tabulated.	0.5<br><br>Identification via books. No vouchers.	0.0<br><br>Descriptive only.	0.5<br><br>Purposive sampling. N=20. Small sample size.	0.0<br><br>Interviews and observation.	0.5<br><br>General interview description.	0.0<br><br>No ethics statement found.	0.0<br><br>Vernacular disease terms used.	3
**53**	**SR_EB_077**	Kajian Etnobotani Tumbuhan Obat oleh Masyarakat Lokal Etnis Batak Mandailing di Desa Tanjung Julu, Kabupaten Mandailing Natal, Sumatra Utara	Silalahi, M., et al.	1.0<br><br>Targeted healers (hatobangon) and general users.	1.0<br><br>Tanjung Julu Village described. Demographics (N=36) detailed.	1.0<br><br>Vouchers identified at Herbarium Bogoriense (LIPI).	1.0<br><br>Calculates UV. Categories defined.	1.0<br><br>Purposive/Snowball. N=36. Adequate for focused study.	0.5<br><br>Participatory observation and interviews.	1.0<br><br>Interview guidelines described.	0.0<br><br>No ethics statement found.	0.0<br><br>General disease terms used.	7

**54**	**SR_EB_ 078**	Etnobotani Tumbuhan Obat Tradisional di Suku Colol Kabupaten Manggarai Timur	Gustina et al.	0.5<br>Criteria listed ("tokoh adat", "expert"), but specific details lacking.	1.0<br>Colol Village described. Demographics (N=5) detailed.	0.5<br>Identification via books/herbarium mentioned. No vouchers cited.	0.0<br>Qualitative/ Descriptive only.	0.5<br>Purposive sampling. N=5 key informants. Very small.	0.0<br>Interviews only.	1.0<br>Questionnaire guide described.	0.0<br>No ethics statement found.	0.0<br>Vernacular disease terms used.	3 5
**55**	**SR_EB_ 079**	Studi Etnobotani Tumbuhan Obat Masyarakat Kelurahan Kembang Paseban Kecamatan Mersam Kabupaten Batanghari	Adriadi, A. et al,	1.0<br>Targeted dukun kampung, elders, and knowledgeable locals.	1.0<br>Kembang Paseban Village described. Demographics (N=20) tabulated.	1.0<br>Specimens identified at Herbarium Universitas Jambi.	1.0<br>Calculates UV. Categories defined.	0.5<br>Purposive/Snowball. N=20. Small sample size.	0.5<br>Field walk ("jelajah") used for verification.	0.5<br>General interview description.	0.0<br>No ethics statement found.	0.0<br>General disease terms used.	5
**56**	**SR_EB_ 080**	Etnobotani tumbuhan obat Suku Ogan di Desa Beringin Dalam, Rambang Kuang, Ogan Ilir	Sarina, A., et al.	1.0<br>Specific criteria: Batra (healers), >40 years old, native.	1.0<br>Beringin Dalam Village described. Demographics (N=7) detailed.	1.0<br>Specimens identified at Herbarium Universitas Andalas (ANDA). High reliability.	0.0<br>Descriptive only (percentages).	0.5<br>Purposive sampling. N=7 healers. Very small.	0.0<br>Observation and interviews.	1.0<br>Questionnaire/Interview guide described.	0.0<br>No ethics statement found.	0.0<br>Vernacular disease terms used.	4 5

**57**	**SR_EB_ 081**	Kajian Etnobotani Tumbuhan Obat Etnis Batak Toba di Desa Sipituhuta, Kec. Pollung, Humbang Hasundutan, Sumatera Utara	Nasution, J., et al.	0.5<br><br>Targeted "knowledgeable community members".	1.0<br><br>Sipituhuta Village described. Demographics (N=30) tabulated.	0.0<br><br>Identification method unclear. No vouchers.	0.0<br><br>Qualitative/ Descriptive only.	0.5<br><br>Purposive sampling. N=30. Reasonable for descriptive study.	0.5<br><br>Observation and interviews.	0.5<br><br>General interview description.	0.0<br><br>No ethics statement found.	0.0<br><br>Vernacular disease terms used.	3
**58**	**SR_EB_ 082**	Etnobotani Pemanfaatan Tumbuhan Obat Oleh Masyarakat Desa Sabah Balau, Kecamatan Tanjung Bintang, Kabupaten Lampung Selatan	Maretta, G., et al.	0.5<br><br>Targeted "local community". Specific selection criteria not detailed.	1.0<br><br>Sabah Balau Village described. Demographics (N=100) tabulated.	0.5<br><br>Identification via books. No vouchers mentioned.	0.0<br><br>Descriptive only (percentages).	1.0<br><br>Purposive sampling. N=100. Robust sample size (using Slovin formula).	0.0<br><br>Interviews and observation.	0.5<br><br>General interview description.	0.0<br><br>No ethics statement found.	0.0<br><br>Vernacular disease terms used.	3 5
**59**	**SR_EB_ 083**	Ethnobotany and Conservation of Wild Edible Fruits in Sumatra: a Case Study in Pesisir Selatan, West Sumatra, Indonesia	Suwardi, A.B., et al.	1.0<br><br>Random sampling from village demographic data. N=393.	1.0<br><br>Pesisir Selatan district. Demographics tabulated.	1.0<br><br>Vouchers identified at Herbarium Universitas Andalas (ANDA). High reliability.	1.0<br><br>Calculates UV, RFC, CI, ICS. Focus is food, but medicinal uses are categorized.	1.0<br><br>Random sampling with large N=393. Robust.	0.0<br><br>Semi-structured interviews. No explicit triangulation mentioned.	1.0<br><br>List of interview questions provided in Table S1.	0.5<br><br>Verbal consent implied/mentioned but ethics board approval not cited.	0.0<br><br>Focus is fruit; medicinal uses mentioned are general.	6 5

**60**	**SR_EB_084**	Ethnomedical Practices and Medicinal Plant Use in Tanjung and Seponjen Villages, Jambi, Indonesia	Wulandari, C., et al.	1.0<br><br>Targeted dukun (healers) and community members.	1.0<br><br>Tanjung and Seponjen Villages described. Demographics (N=30 each) tabulated.	0.0<br><br>Identification method unclear. No vouchers.	1.0<br><br>Calculates ICS. Categories defined.	1.0<br><br>Purposive sampling. N=60 (30/village). Reasonable size.	0.5<br><br>Interviews and field observation.	0.5<br><br>General description of interview guide.	0.0<br><br>No ethics statement found.	0.0<br><br>Vernacular disease terms used.	5
**61**	**SR_EB_085**	Ethnobotanical study of Kaili Inde tribe in Central Sulawesi Indonesia	Fathurrahman, F., et al.	1.0<br><br>Key informants (leaders, healers) and general respondents.	1.0<br><br>Mantikole Village described. Demographics (N=8) detailed.	1.0<br><br>Voucher specimens identified at Herbarium Celebense (UNTAD).	1.0<br><br>Calculates ICS. Use categories defined (Purwanto, 2004).	0.5<br><br>Purposive. N=8 is very small for a general ethnobotanical survey.	0.5<br><br>Transect walk used to verify plant existence.	0.5<br><br>General interview description.	0.0<br><br>No ethics statement found.	0.0<br><br>General disease terms used.	5
**62**	**SR_EB_086**	Etnobotani Tumbuhan Obat Masyarakat Subetnis Tonsawang di Kabupaten Minahasa Tenggara Provinsi Sulawesi Utara	Mamahani, A.F., et al.	0.5<br><br>Criteria implied but not explicitly detailed.	1.0<br><br>Minahasa Tenggara. Demographics (N=80) tabulated.	0.0<br><br>Explorative survey. No vouchers mentioned.	0.0<br><br>Descriptive only (percentages).	1.0<br><br>Purposive sampling. N=80. Robust sample size.	0.0<br><br>Interviews only.	0.5<br><br>General interview description.	0.0<br><br>No ethics statement found.	0.0<br><br>Vernacular disease terms used.	3

**63**	**SR_EB_087**	Jenis Tumbuhan yang Digunakan sebagai Obat Tradisional di Daerah Eks-Karesidenan Surakarta	Dewantari, R. et al.	0.5<br><br>Criteria: "Community members who use traditional medicine".	0.5<br><br>Ex-Residency of Surakarta (broad area). Respondent demographics not detailed.	0.0<br><br>Identification method unclear. No vouchers.	0.0<br><br>Descriptive only.	0.5<br><br>Survey method. Sample size N not clearly stated in abstract/methods snippet.	0.0<br><br>Interviews only.	0.5<br><br>General interview description.	0.0<br><br>No ethics statement found.	0.0<br><br>General terms used.	1 5
**64**	**SR_EB_088**	Studi Etnobotani Tumbuhan Obat di Kawasan Sekitar Danau Buyan-Tamblingan, Bali	Oktavia, G.A.E., et al.	1.0<br><br>Specific criteria: Balian (traditional healers) and elders.	1.0<br><br>Buyan-Tamblingan area. Respondent demographics (N=5) detailed.	1.0<br><br>Vouchers identified at Bali Botanic Garden. High reliability.	0.0<br><br>Qualitative/ Descriptive only.	0.5<br><br>Purposive sampling. N=5 key informants. Very small sample size.	0.0<br><br>Observation and interviews.	0.5<br><br>General interview description.	0.0<br><br>No ethics statement found.	0.0<br><br>Vernacular disease terms used.	4
**65**	**SR_EB_089**	Kajian Etnobotani dan Bioaktifitas Tumbuhan Obat Kabupaten Tapanuli Utara	Sinaga, A.H. and Manalu, A.I	0.5<br><br>Targeted "traditional healers". Specific criteria not detailed.	1.0<br><br>Tapanuli Utara (3 villages). Demographics (N=3) tabulated.	1.0<br><br>Herbarium specimens prepared. Identification method implied (standard).	0.5<br><br>Calculates UV, RFC, RI. However, data presentation is limited.	0.5<br><br>Purposive sampling. N=3 healers. Very small.	0.0<br><br>Interviews and field survey.	0.5<br><br>General interview description.	0.0<br><br>No ethics statement found.	0.0<br><br>General disease terms used.	4

**66**	**SR_EB_ 090**	Kajian Etnobotani Masyarakat Kampung Adat Dukuh Kabupaten Garut, Jawa Barat (Ethnobotanical Study of Local People at Dukuh Cultural Village Garut Regency, West Java)	Hidayat, S., et al.	1.0<br><br>Targeted Kuncen, traditional leaders, and local community.	1.0<br><br>Kampung Adat Dukuh described. Demographics (N=30) detailed.	1.0<br><br>Vouchers identified at Herbarium Bogoriense (LIPI). High reliability.	0.5<br><br>Calculates UV, ICS. Use categories defined.	1.0<br><br>Purposive/Snowball. N=30. Adequate for specific cultural enclave.	0.5<br><br>Observation, interviews, and participation.	0.5<br><br>General interview description.	0.0<br><br>No ethics statement found.	0.0<br><br>Vernacular disease terms used.	5 5
**67**	**SR_EB_ 091**	Potensi Etnobotani Masyarakat Desa Sekitar Hutan (Studi Kasus di Desa Tamanjaya, Kecamatan Sumur, Kabupaten Pandeglang, Banten)	Asmemare, K. et al.	0.5<br><br>Targeted "community members". Criteria not explicitly detailed.	1.0<br><br>Tamanjaya Village described. Demographics (N=86) tabulated.	0.0<br><br>Identification method unclear. No vouchers.	0.0<br><br>Descriptive only (percentages).	1.0<br><br>Purposive sampling. N=86. Robust sample size.	0.0<br><br>Observation and interviews.	1.0<br><br>Questionnaire used.	0.0<br><br>No ethics statement found.	0.0<br><br>General disease terms used.	3 5

**68**	**SR_EB_092**	Etnobotani Tanaman Obat di Kecamatan Nangapanda Kabupaten Ende Nusa Tenggara Timur	Tima, M.T., et al.	0.5<br>Criteria implied but not explicitly detailed.	1.0<br>Nangapanda District (3 villages). Demographics (N=90) tabulated.	0.0<br>Identification method unclear. No vouchers.	0.0<br>Descriptive only (percentages).	1.0<br>Purposive sampling. N=90. Robust sample size.	0.5<br>Interviews and "track" (field walk) to verify data.	1.0<br>Questionnaire used.	0.0<br>No ethics statement found.	0.0<br>General disease terms used.	4
**69**	**SR_EB_094**	Etnobotani Tumbuhan Obat oleh Etnis Dayak Meratus di Desa Gunung Riut Kabupaten Balangan Kalimantan Selatan	Wathan, N.,et al.	1.0<br>Criteria: Dayak Meratus ethnicity, accustomed to using/treating with plants, willing.	1.0<br>Gunung Riut Village described. Informant profiles (N=5) described.	0.5<br>Identification via literature/online tools. No voucher specimens mentioned.	0.0<br>Descriptive only (percentages).	0.5<br>Purposive sampling. N=5 key informants. Very small.	0.5<br>Data validity check mentioned (re-contacting informants).	1.0<br>Questionnaire used.	0.0<br>No ethics statement found.	0.0<br>Vernacular disease terms used.	4 5
**70**	**SR_EB_095**	Studi Etnobotani Jenis Tumbuhan Obat pada Masyarakat Kecamatan Beutong Ateuh Banggalang, Kabupaten Nagan Raya	Nurjannah et al.	1.0<br>Specific criteria: Key (healers) vs Non-key (users, >35 years, >5 years resident).	1.0<br>4 villages in Beutong Ateuh. Sample size calculation (5% of households) detailed (N=33).	0.0<br>Observation/documentation only. No identification method cited.	0.0<br>Descriptive only.	1.0<br>Purposive/Snowball + Quota (5% of KK). Good justification.	0.5<br>Uses Participatory Rural Appraisal (PRA).	1.0<br>Questionnaire used.	0.0<br>No ethics statement found.	0.0<br>Vernacular disease terms used.	5 5

**71**	**SR_EB_096**	Studi Etnobotani Pemanfaatan Tanaman Obat Masyarakat Desa Sabulakoa Kabupaten Konawe Selatan	Haris, R.N.H., et al.	1.0<br><br>Criteria: Experience, knowledge of mixing/usage, willing (Inclusion/Exclusion defined).	1.0<br><br>Sabulakoa Village described. Demographics (N=30) detailed.	1.0<br><br>Determination at Biology Study Program, UHO. High reliability.	0.0<br><br>Descriptive tables.	1.0<br><br>Purposive sampling. N=30. Adequate for village study.	0.5<br><br>Interviews and field observation.	0.5<br><br>General interview description.	0.0<br><br>No ethics statement found.	0.0<br><br>General disease terms used.	6	5
**72**	**SR_EB_097**	Studi Etnobotani Tanaman Berkhasiat Obat Pada Etnis Ende, Lio, Nataia, Dhawe Pulau Flores Provinsi Nusa Tenggara Timur	Fajrawati, K	1.0<br><br>Criteria: >25 years old.	1.0<br><br>4 ethnicities (Ende, Lio, Nataia, Dhawe). N=77. Demographics detailed.	0.0<br><br>Identification by matching local names or photos. No vouchers.	0.0<br><br>Descriptive only.	1.0<br><br>Purposive/Snowball. N=77 (approx 20/ethnic). Robust size.	0.0<br><br>Observation and interviews.	0.5<br><br>General methodology description.	0.0<br><br>No ethics statement found.	0.0<br><br>General disease terms used.	4	5
**73**	**SR_EB_098**	Identifikasi Etnobotani Tanaman Obat yang Dimanfaatkan oleh Masyarakat Kecamatan Sempor Kabupaten Kebumen	Widiastuti, T.C.,et al.	1.0<br><br>Criteria: Resident, long-time user, has plants at home, communicative, willing.	1.0<br><br>5 areas in Sempor District. N=50. Demographics detailed.	0.0<br><br>Survey method. No identification details provided.	0.0<br><br>Descriptive categories only.	1.0<br><br>Random sampling (stated, though description sounds purposive/quota). N=50. Reasonable.	0.5<br><br>Survey and direct field observation.	1.0<br><br>Questionnaire items listed.	0.0<br><br>No ethics statement found.	0.0<br><br>General disease terms used.	5	5

**74**	**SR_EB_099**	Ethnobotanical Study of Medicinal Plants in Taro Village, Gianyar, Bali	Buu, A.W., et al.	0.0<br>Criteria unclear; mentions "healers and members" but selection process is vague.	1.0<br>Taro Village described. Demographics not detailed.	0.5<br>Identification via comparison, images, experts. No voucher numbers cited.	0.0<br>Descriptive tables only. No quantitative indices.	0.0<br>"Random sampling" stated but methodology suggests purposive/snowball. N not clearly justified.	0.0<br>Interviews and observation.	0.0<br>No instrument details.	0.0<br>No ethics statement found.	0.0<br>Vernacular terms used.	1 5
**75**	**SR_EB_100**	Kajian Etnobotani Tumbuhan Obat oleh Masyarakat Dayak Deah Desa Pangelak Kecamatan Upau Kabupaten Tabalong Provinsi Kalimantan Selatan	Tampublon, A.O., et al.	1.0<br>Specific criteria: tokoh adat, batra (healers).	1.0<br>Pangelak Village described. Respondent profiles (N=4) detailed.	0.0<br>Identification method unclear. No vouchers.	0.0<br>Descriptive only (percentages).	0.5<br>Purposive sampling. N=4 key informants. Very small.	0.0<br>In-depth interviews.	1.0<br>Questionnaire used.	0.0<br>No ethics statement found.	0.0<br>Vernacular terms used.	3 5
**76**	**SR_EB_101**	Studi etnobotani tumbuhan obat di Kampung Adat Cireundeu Kota Cimahi Jawa Barat	Nurani, S. and Cahyanto, T.	1.0<br>Criteria: Native residents, knowledgeable, willing.	1.0<br>Cireundeu Village mapped. Demographics (N=15) detailed.	0.0<br>No identification method or voucher specimens mentioned.	0.0<br>Descriptive only (percentages).	0.5<br>Purposive sampling. N=15. Small sample size.	0.5<br>Explorative survey + in-depth interviews (triangulation).	0.5<br>General interview description.	0.0<br>No ethics statement found.	0.0<br>General disease terms used.	3 5

**77**	**SR_EB_ 102**	KAJIAN ETNOBOTANI TUMBUHAN OBAT OLEH MASYARAKAT BALI KABUPATEN BARITO KUALA (Ethnobotanical Study of Medicinal Plants by the Community of Balinese, Barito Kuala District)	Ninawati et al.	1.0<br><br>Criteria: Knowledgeable about plants and traditional medicine use.	1.0<br><br>Barambai Kolam Kanan Village described. Demographics (N=15) mentioned.	0.0<br><br>Identification method unclear. No vouchers.	0.0<br><br>Descriptive only.	0.5<br><br>Snowball sampling. N=15. Small sample size.	0.5<br><br>Field observation + interviews.	1.0<br><br>Questionnaire used.	0.0<br><br>No ethics statement found.	0.0<br><br>General disease terms used.	4
**78**	**SR_EB_ 103**	Etnobotani Tumbuhan Liar sebagai Obat Herbal di Lingkungan Perumahan Grand Tamansari 3 Kabupaten Bekasi	Safitri, D.S., et al.	1.0<br><br>Criteria: Can explain plant knowledge, used wild plants.	1.0<br><br>Housing complex mapped. Respondent profiles (N=7) detailed.	0.0<br><br>Identification via literature/apps likely (citations in table). No vouchers.	0.0<br><br>Descriptive only.	0.5<br><br>Purposive sampling. N=7. Very small sample size.	1.0<br><br>Triangulation explicitly mentioned (observation, interview, documentation).	1.0<br><br>Observation sheet and interview guide used.	0.0<br><br>No ethics statement found.	0.0<br><br>General disease terms used.	5

**79**	**SR_EB_ 104**	Studi Etnobotani Tanaman Obat Tradisional pada Masyarakat di Desa Orahili Kecamatan Sirombu Kabupaten Nias Barat	Daeli, D.Y.	0.5<br><br>Criteria implied (knowledgeable) but not explicitly detailed.	1.0<br><br>Orahili Village described. N=10 informants.	0.0<br><br>Identification method unclear. No vouchers.	0.0<br><br>Qualitative/ Descriptive only.	0.5<br><br>Purposive sampling. N=10. Very small.	0.0<br><br>Interviews, observation, documentation.	0.5<br><br>General method description.	0.0<br><br>No ethics statement found.	0.0<br><br>Vernacular disease terms used.	3
**80**	**SR_EB_ 105**	Kajian Etnobotani Tanaman Obat di Pasar Dayak Kalimantan Utara (Ethnobotanical Study of Medicinal Plants in the Dayak Market of North Kalimantan)	Suciyati, A. and Retnaningati	1.0<br><br>Specific criteria: herbal traders and spice sellers in Pasar Dayak.	1.0<br><br>Pasar Dayak, Tarakan. Setting described. Informant N implied (traders).	0.0<br><br>Identification via literature/apps likely. No vouchers.	0.0<br><br>Descriptive only.	0.5<br><br>Descriptive survey. N not explicitly stated in abstract/methods (implied small).	0.5<br><br>Observation + interviews + literature study (triangulation).	0.5<br><br>General method description.	0.0<br><br>No ethics statement found.	0.0<br><br>General disease terms used.	3

**81**	**SR_EB_106**	Kajian Etnobotani Tumbuhan Obat Suku Dayak Lundayeh di Desa Kaliamok Kecamatan Malinau Utara Kabupaten Malinau sebagai Booklet untuk Masyarakat	Setiawan, A. et al.	0.5<br><br>Targeted "community" for interviews and "experts" for validation.	1.0<br><br>Kaliamok Village. N=20 interviewees + 36 test subjects.	0.5<br><br>Identification via books (Van Steenis etc.). No vouchers.	0.0<br><br>R&D study focus (booklet). Descriptive ethnobotany.	1.0<br><br>Purposive sampling (N=20). Adequate for initial data collection for booklet.	0.0<br><br>Interviews only for ethnobotany data.	1.0<br><br>Interview, questionnaire, and validation sheets described.	0.0<br><br>No ethics statement found.	0.0<br><br>General disease terms used.	4
**82**	**SR_EB_107**	Studi Etnobotani Tumbuhan Obat dalam Masyarakat Adat Tigo Luhah Tanah Sekudung Siulak Kabupaten Kerinci	Santosa, T.A., et al.	1.0<br><br>Specific criteria: dukun kampung and knowledgeable community members.	1.0<br><br>Mukai Hilir Village. Demographics (N=10) detailed (8 community, 2 healers).	0.0<br><br>Identification method unclear (observation mentioned). No vouchers.	0.0<br><br>Descriptive only.	0.5<br><br>Purposive sampling. N=10. Very small.	0.5<br><br>Triangulation explicitly mentioned as validity technique.	0.5<br><br>Questionnaire/Interview mentioned.	0.0<br><br>No ethics statement found.	0.0<br><br>Vernacular disease terms used.	3

**83**	**SR_EB_ 108**	Kajian Etnobotani Tanaman Berkhasiat Obat di Desa Cipacing Kecamatan Jatinangor Kabupaten Sumedang Jawa Barat	Maulidina, I. and Cahyanto, T.	1.0<br><br>Criteria: Know and use medicinal plants.	1.0<br><br>Cipacing Village described. Demographics (N=30) detailed.	0.5<br><br>Identification via books. No vouchers.	0.0<br><br>Descriptive only (percentages).	1.0<br><br>Purposive sampling. N=30. Adequate for descriptive study.	0.0<br><br>Interviews and observation.	0.5<br><br>Semi-structured interview mentioned.	0.0<br><br>No ethics statement found.	0.0<br><br>General disease terms used.		4
**84**	**SR_EB_ 109**	Studi Etnobotani Tumbuhan Obat-Obatan Tradisional Suku Melayu di Desa Mungguk Kecamatan Sekadau Hilir Kabupaten Sekadau	Selpi et al.	1.0<br><br>Specific criteria: Dukun, elders, native Malay residents, knowledgeable.	1.0<br><br>Mungguk Village described. N=10 informants.	1.0<br><br>Identification via books/journals. Herbarium made (dry).	1.0<br><br>Calculates SUV. Categories defined.	0.5<br><br>Snowball sampling. N=10. Very small sample size.	0.0<br><br>Semi-structured interviews.	1.0<br><br>Questionnaire items listed.	0.0<br><br>No ethics statement found.	0.0<br><br>Vernacular disease terms used.	5	5
**85**	**SR_EB_ 110**	Etnobotani Tumbuhan Obat Pada Masyarakat Desa Teluk Rendah di Kecamatan Tebo Ilir Kabupaten Tebo	Adriadi, A., et al.	1.0<br><br>Criteria: Key (healers, elders) and Non-key (users).	1.0<br><br>Teluk Rendah Ilir Village described. N=23 (4 key + 19 non-key).	0.0<br><br>Observation/interview only. No identification method cited.	1.0<br><br>Calculates PPV.	1.0<br><br>Purposive sampling. N=23. Reasonable for village study.	0.5<br><br>In-depth interviews and observation.	0.5<br><br>General description of interview.	0.0<br><br>No ethics statement found.	0.0<br><br>Vernacular disease terms used.		5

**86**	**SR_EB_ 111**	Etnobotani Tumbuhan Obat oleh Masyarakat Desa Sungai Ulu Kabupaten Natuna	Suri, L.A.,et al.	0.0<br>Snowball sampling mentioned, but specific criteria not detailed.	1.0<br>Sungai Ulu Village described. N=55 informants.	1.0<br>Specimens collected for herbarium to assist ID.	0.0<br>Descriptive only.	1.0<br>Snowball sampling. N=55. Robust sample size.	1.0<br>Triangulation explicitly used (interview, observation, documentation).	1.0<br>Interview sheet modified from Ristoja.	0.0<br>No ethics statement found.	0.0<br>General disease terms used.		5
**87**	**SR_EB_ 112**	Studi Etnobotani Pemanfaatan Tumbuhan Obat Tradisional oleh Masyarakat Nagari Sijunjung / Ethnobotanical study on traditional medicinal plants by community in Nagari Sijunjung	Andania, M.M., et al.	1.0<br>Criteria: Native citizens of Nagari Sijunjung.	1.0<br>Nagari Sijunjung described. N=39 respondents.	0.0<br>Observation /documentation. No specific ID method cited.	0.0<br>Descriptive only.	1.0<br>Random/general interview. N=39. Reasonable.	0.5<br>Field observation + interviews.	1.0<br>Questionnaire used.	0.0<br>No ethics statement found.	0.0<br>General disease terms used.	4	5

**88**	**SR_EB_ 113**	Kajian Etnobotani dan Etnofarmakologi Tanaman Obat yang Dimanfaatkan Masyarakat Pesisir Gugus Kepulauan Kei, Kota Tual, Maluku	Agustin, R.D., et al.	0.5<br><br>Probability proportional to size sampling. Criteria not explicitly detailed.	1.0<br><br>Kota Tual (10 villages). N=64 informants.	0.0<br><br>Identification method unclear.	1.0<br><br>Calculates Frequency of Citation (FC).	1.0<br><br>Probability sampling. N=64. Robust.	0.0<br><br>Interviews only.	0.5<br><br>General description of interview.	0.0<br><br>No ethics statement found.	0.0<br><br>General disease terms used.	4
**89**	**SR_EB_ 114**	Studi Etnobotani Penggunaan Tanaman Berkhasiat Obat pada Masyarakat di Kecamatan Mirit Kabupaten Kebumen Jawa Tengah	Widodo, R.C., et al.	1.0<br><br>Criteria: Indigenous, >40 years old, know/use plants.	1.0<br><br>Mirit District described. N=100 respondents.	1.0<br><br>Specimens identified at Laboratorium Biologi Farmasi UMP.	0.0<br><br>Descriptive percentages only.	1.0<br><br>Purposive sampling. N=100. Robust sample size.	0.0<br><br>Interviews only.	1.0<br><br>Questionnaire used.	1.0<br><br>Research permit mentioned.	0.0<br><br>General disease terms used.	6
**90**	**SR_EB_ 115**	Etnobotani Tumbuhan Obat Oleh Masyarakat Bolaang Mongondow	Pangemanan, E.F.S., et al.	1.0<br><br>Criteria: Age >30, married, local resident, knowledgeable.	1.0<br><br>Bolaang Mongondow. Demographics (N=50) detailed.	0.0<br><br>Identification via literature/apps likely. No vouchers.	0.0<br><br>Descriptive only.	1.0<br><br>Purposive sampling. N=50. Robust sample size.	0.5<br><br>Field observation + interviews.	1.0<br><br>Questionnaire used.	0.0<br><br>No ethics statement found.	0.0<br><br>General disease terms used.	4 5

**91**	**SR_EB_116**	Ethnobotany of Medicinal Plants in Patemon Village, Tengaran, Semarang District	Jaya, S. and Suprihati	1.0<br>Key informants (hamlet heads, healers) + Supporting (farmers).	1.0<br>Patemon Village described. Informant profiles (N=15) detailed.	0.0<br>Identification method unclear. No vouchers.	1.0<br>Calculates RFC.	1.0<br>Snowball sampling. N=15 (10 key + 5 supporting). Adequate for qualitative study.	0.0<br>Interviews only.	0.5<br>General description of interview.	0.0<br>No ethics statement found.	0.0<br>General disease terms used.	4	5
